# Predicting COVID-19 booster immunogenicity against future SARS-CoV-2 variants and the benefits of vaccine updates

**DOI:** 10.1038/s41467-024-52194-9

**Published:** 2024-09-27

**Authors:** Deborah Cromer, Arnold Reynaldi, Ainslie Mitchell, Timothy E. Schlub, Jennifer A. Juno, Adam K. Wheatley, Stephen J. Kent, David S. Khoury, Miles P. Davenport

**Affiliations:** 1 Kirby Institute, University of New South Wales, Sydney, NSW Australia; 2 Sydney School of Public Health, Faculty of Medicine and Health, University of Sydney, Sydney, NSW Australia; 3 Department of Microbiology and Immunology, University of Melbourne at the Peter Doherty Institute for Infection and Immunity, Melbourne, VIC Australia; 4 Melbourne Sexual Health Centre and Department of Infectious Diseases, Alfred Hospital and Central Clinical School, Monash University, Melbourne, VIC Australia

**Keywords:** Viral infection, Vaccines, Epidemiology, SARS-CoV-2

## Abstract

The ongoing evolution of the SARS-CoV-2 virus has led to a move to update vaccine antigens in 2022 and 2023. These updated antigens were chosen and approved based largely on in vitro neutralisation titres against recent SARS-CoV-2 variants. However, unavoidable delays in vaccine manufacture and distribution meant that the updated booster vaccine was no longer well-matched to the circulating SARS-CoV-2 variant by the time of its deployment. Understanding whether the updating of booster vaccine antigens improves immune responses to subsequent SARS-CoV-2 circulating variants is a major priority in justifying future vaccine updates. Here we analyse all available data on the immunogenicity of variants containing SARS-CoV-2 vaccines and their ability to neutralise later circulating SARS-CoV-2 variants. We find that updated booster antigens give a 1.4-fold [95% CI: 1.07–1.82] greater increase in neutralising antibody levels when compared with a historical vaccine immunogen. We then use this to predict the relative protection that can be expected from an updated vaccine even when the circulating variant has evolved away from the updated vaccine immunogen. These findings help inform the rollout of future booster vaccination programmes.

## Abstract (web summary)

SARS-CoV-2 vaccines have been updated to include antigens from new variants. Here, the authors analyse data from published studies to investigate whether updating vaccines provides additional immunity to subsequent emerging strains compared to using original vaccines.

## Introduction

The continual emergence of novel SARS-CoV-2 variants has driven vaccine manufacturers and regulatory bodies to update COVID-19 vaccine immunogens^1,2^. Ideally, the choice of which vaccine to deploy would be informed by vaccine efficacy trials against the relevant circulating SARS-CoV-2 strains. However, the decision of which immunogen to include in a vaccine occurs well before a vaccine’s eventual approval, manufacture, and deployment, and therefore randomised controlled trials are not possible in the time required to inform strain selection decisions^3^. As a result, the assessment and comparison of different updated vaccine immunogens has been made based largely on neutralising antibody titres after vaccination, most commonly against the vaccine immunogen^4–7^. Since continued evolution and neutralisation escape can occur before an updated vaccine is widely administered, in general, the circulating variant at the time of vaccine deployment no longer matches the immunogen that was included in the vaccine and against which the vaccine was assessed^8^.

The advantage of an update to a vaccine immunogen therefore must be assessed in the context of an anticipated mismatch between the vaccine and the circulating strains. The relative benefits of updating a vaccine immunogen are thought to be dependent on the antigenic relationships between the historical, contemporaneous and future circulating variants. That is, the infection and vaccination history of an individual are thought to contribute to their current cross-recognition profile of different variant strains^9^. Further, boosting with a variant antigen leads to increased recognition of the vaccine strain (and antigenically similar strains)^10^. How effective this is at improving responses to a future variant is thought to be dependent on the similarity between the booster antigen and a future circulating variant (antigenic distance)^11^. However, this relationship to the future circulating variant is inherently unknowable at the time when the decision on which immunogen to include in a vaccine needs to be made. An alternative approach is to look to past experience to inform the likely future outcomes. That is, we can look to past examples and existing data to ask what (average) advantage updating the vaccine immunogen gave to recognition of a future variant.

**Fig. 1:**
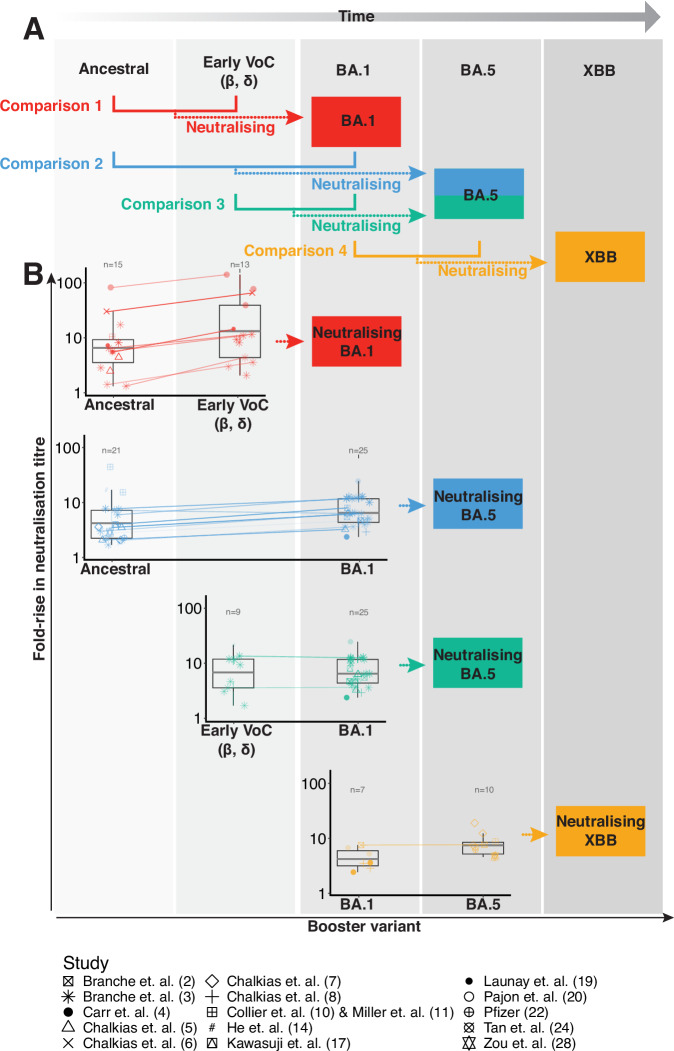
Possible comparisons between different SARS-CoV-2 vaccine immunogens. **A** Chronology of the appearance of different SARS-CoV-2 variants and associated vaccine immunogens, and the comparisons made between different vaccine immunogens to induce neutralisation against future variants (coloured arrows). **B** Data extracted from identified papers could contribute to each of the comparisons outlined in (**A**). Lines connect paired cohorts from the same study. The centre lines of the boxes show the median; box limits show the interquartile range; and whiskers show the range of the data (outliers that are more than 1.5 times the interquartile ranges from the box edge are not shown).

In this work, we identified all available comparative studies of different vaccine immunogens and considered the reported in vitro neutralisation titres against a variant that primarily circulated during/after deployment of the vaccine (defined in Fig. 1). We compared pairs of potential immunogens at a given time, one designated as ‘old’ and one as ‘updated’, and assessed their immunogenicity against a future variant (i.e. against a variant that circulated chronologically after the variant immunogen that was used in either the old or updated vaccine). This allowed us to identify the degree to which, for a given variant wave, an ‘updated’ booster provided a better boost to the neutralising antibody titres of a future variant than an ‘old’ booster. We then used this data, and the established relationship between neutralising antibody response and protection from COVID-19, to predict the additional protection from COVID-19 disease that could be achieved by using an updated vaccine immunogen.

## Results

### Identification of data from relevant studies

We identified 28 studies^4,5,10,12–36^ in which neutralising antibody titres were measured against future variants following variant-containing booster vaccination (Supplementary Table 1). From these, we identified studies that considered in vitro neutralisation titres against BA.1, BA.5 and XBB variants, following booster vaccination with vaccines containing Ancestral, Beta, Delta, BA.1, BA.5 and XBB immunogens. Of these, 18 studies reported any pre-boost neutralisation titres (though in four of these studies, pre-boost titres were not reported for all immunogen/variant combinations) and 10 studies only reported post-boost titres. Two studies analysed responses to a single relevant immunogen-containing booster, 20 compared neutralising antibodies after two different immunogen-containing boosters, and 6 compared responses after three or more immunogen-containing boosters.

### Immunogen comparisons

Our aim was to consider whether boosting with an updated immunogen confers higher neutralising antibody titres to a future (at the time unknown) variant than boosting with an older immunogen. We identified four such comparisons that could be made from the available data, and these are shown by the coloured lines in Fig. 1A and are detailed in Supplementary Table 2.

Of the 28 studies we identified, only two studies^10,12^ contained data that could be used to contribute to all four comparisons, however, no study contained data for both the old and updated immunogens across all four comparisons. In addition, 1 study could not be used in our analysis as it did not include titres after one of the relevant immunogens^19^, leaving 27 studies that could contribute to one or more of the comparisons identified in Fig. 1A. Table 1 shows the number of studies we identified that could contribute to each comparison, and also highlights how many of these included pre-boost neutralisation titres, which could be used to calculate the fold-change in neutralisation titres after boosting. The fold changes from the 16 studies that included pre-boost neutralisation titres are depicted in Fig. 1B.

**Table 1:** Number of studies reporting neutralisation titres against a future variant for each of the four possible comparisons

Comparison	Comp 1 ancestral vs early VoC	Comp 2 ancestral vs BA.1	Comp 3 early VoC vs BA.1	Comp 4 BA.1 vs BA.5
**Neutralisation titres against:**	**BA.1**	**BA.5**	**BA.5**	**XBB**
Either old or updated immunogens	15 (7)	24 (13)	11 (8)	15 (7)
Both old and updated immunogens	5 (4)	5 (4)	1 (1)	3 (1)
Benefit of an update to fold rise in neutralisation titres [95% CI]	1.52 [1.27–1.81]	1.68 [1.45–1.94]	1.11 [0.88–1.41]	1.19 [0.84–1.68]

Studies are split by those with data for either the old or updated immunogen and those with data for both the old and updated immunogens. Numbers in brackets give the number of studies that also included pre-boost neutralisation titres, and could therefore be included when modelling the fold-rise in neutralisation titres after boosting.

### Updated immunogens provide a greater boost to a future variant

We first investigated the relative benefit of an updated booster versus an older booster on neutralisation titres against the future variant (across the comparisons defined in Fig. 1A). We constructed a mixed effects linear regression model that accounted for between-study heterogeneity and the differences in the sizes of the cohorts and incorporated data from 16 (of 27) studies that provided both pre and post boost neutralisation data. We found that updated antigens consistently predicted greater increases in neutralisation against the relevant variant.

The advantage of an updated booster ranged from a 1.11 to 1.68-fold increase in the fold-rise in neutralising antibody titres (Table 1). Considering all the data identified from studies that could contribute to the comparisons (56 cohorts from 16 studies), we found that the neutralisation titres against the specified future variant were, on average, 1.57-fold [95% CI: 1.45–1.69] greater after boosting with the updated immunogen compared to the older immunogen.

The calculation above is subject to potential confounders. In particular, the comparisons being made (number 1, 2, 3, or 4 from Fig. 1A) occurred in the context of different immune histories (e.g. different numbers of prior vaccinations and/or infections, Supplementary Figs. 1 and 2). Therefore, we added these factors as co-variates in our model, the results of which are presented in Table 2. We found that consistent with previous work^37^, the overall level of boosting (of both old and updated vaccines) declined with an increasing number of prior exposures (by 2.5-fold per additional exposure [95% CI: 2.1–3.1]. However, even after accounting for the number of prior exposures, boosting with an updated immunogen was estimated to give a 1.40-fold better boost than boosting with an older immunogen ([95% CI = 1.06–1.84], *p* = 0.02. That is, on average, neutralisation titres to the future variant were boosted 40% higher after an updated immunogen boost, than after an older immunogen boost.

**Table 2:** Parameter values and their interpretations for the mixed effects model

Dependant variable	Parameter	Value, (95% CI), *p*-value	Interpretation
Fold rise in neutralisation titres after boosting	Intercept	1.245 (1.067–1.423), *p* < 0.0001	An initial boost with an older immunogen gives a 10^1.245^ = 17.6-fold rise in titre
	Immunogen (updated vs older)	0.145 (0.025–0.265), *p* = 0.018	Updated immunogen provides 10^0.145^ = 1.4-fold greater rise in titre than older immunogen.
	Exposure number	−0.402 (−0.486 to −0.318), *p* < 0.0001	Each additional prior exposure reduces the fold rise by 10^0.402^ = 2.5-fold.

The mixed effects model is described in Equation S2, and predicts the fold-rise in neutralisation titres following boosting. It fits the data shown in Supplementary Fig. 1A. Values in the table are determined using the glmmTMB function from the R package glmmTMB, in which *p*-values are obtained from a two-sided Wald test.

To assess the robustness of these observations we performed a range of sensitivity analyses. To confirm that cohorts with mixed prior infection status were not impacting our results, we repeated our analysis considering only uninfected cohorts or cohorts with homogeneous prior infection status (Supplementary Table 3 and Supplementary Table 4, respectively). We found that in both cases an updated immunogen also provided a greater boost to neutralisation titres (1.55 [95% CI: 1.12–2.13] and 1.58 [95% CI: 1.17–2.12], respectively).

Another potential confounder is that the early VoC immunogens (e.g. Beta and Delta-containing vaccines) were never actually deployed in the population against COVID-19, unlike the other boosters immunogens we consider. To confirm that the comparisons incorporating early VoC vaccine immunogens were not biasing our results, we repeated the analysis excluding any comparison that involved an early VoC as the most recent immunogen in the booster (ie: considering only comparisons 2 and 4). We found an almost identical benefit from the use of updated booster immunogen when excluding these data (1.42-fold [95% CI: 1.07–1.88] benefit of the updated immunogen, Supplementary Table 5).

Decisions on which immunogen to include in a vaccine are generally made on the basis of fold-rises in neutralisation titre after boosting (as described above). However, under 60% (16/27) of the studies that we identified reported both pre- and post-boost titres necessary to calculate fold-rise in titre for the immunogen/variant combinations required, and so only 16 studies contributed to the results presented above. Therefore, to confirm that the actual neutralisation titres were improved by updating the vaccine immunogen (and not just the fold increase in neutralisation titres), we repeated the above analysis on the absolute neutralisation titres to a future variant reported after boosting (Supplementary Fig. 3), which allowed us to incorporate the data from all 27 studies. We found that the absolute neutralisation titres after boosting with an updated immunogen were estimated to be 1.52-fold [95% CI: 1.30–1.77] higher than after boosting with an older immunogen (Supplementary Table 6).

Together, this demonstrates that booster vaccines containing chronologically more recent variant immunogens, have to date provided better neutralisation responses to the relevant future variant wave that circulated after their deployment.

### The optimal frequency for updating booster immunogens

The analysis above considers an updated booster immunogen against a recent alternative booster regimen. To date, the booster immunogens have been updated annually^1,2^. However, it is possible to ask whether less frequent updating would achieve similar results. For example, in late 2022/early 2023, the XBB family variants were the dominant circulating strains, and there were three potential booster immunogens available for use; the original ancestral booster (dominant until mid-2021), an ‘older’ BA.1 booster (dominant late 2021/early 2022), and an ‘updated’ BA.5 booster (dominant mid-2022). Using the available data for all three of these booster immunogens, we can ask whether repeated immunogen updates provided continued improvement to the boost in neutralisation titres.

**Fig. 2:**
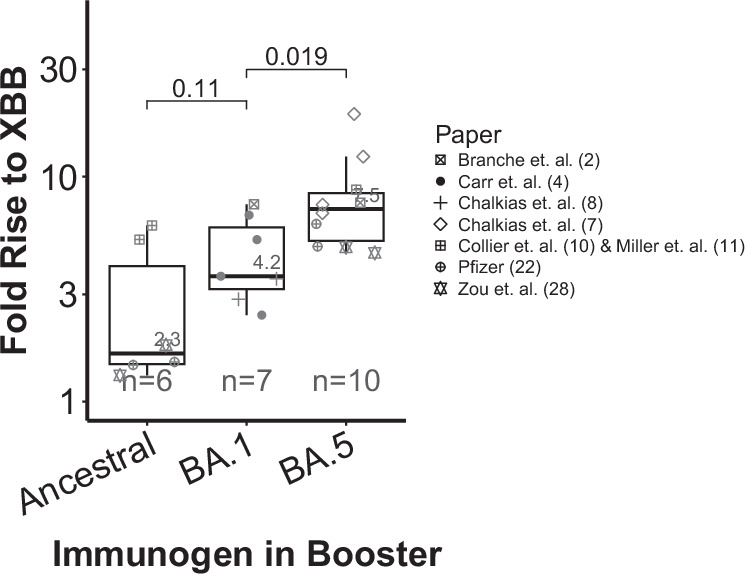
Fold rise in neutralisation titres to the XBB family variants after boosting with vaccines containing different booster immunogens. Small numbers show the geometric mean of the fold rises for each immunogen. Comparisons across the top show *p*-values from two-sided unpaired *t*-tests. The centre lines of the boxes show the median; box limits show the interquartile range; and whiskers show the range of the data (excluding outliers that are more than 1.5 times the interquartile ranges from the box edge).

When comparing in vitro neutralisation titres against XBB family variants we find that the update from the ancestral booster to a BA.1 booster improved the obtained boost in neutralisation titres, and the further update from the BA.1 to a BA.5 vaccine immunogen gave an even better boost (Fig. 2) From this, we can see that both the BA.1 and BA.5 variant immunogens gave a superior boost to XBB neutralisation titres than did the ancestral immunogen (Fig. 2, in line with our previous work^38^) and in addition, that the updated BA.5 immunogen gave a better boost than the older BA.1 immunogen.

Together these observations suggest that if we were to stop updating booster antigens, then the current boosters would give a lower and lower boost to successive future variants. This suggests continued benefit for annual updating of the booster immunogen.

### Predicting the clinical benefit of updating the vaccine immunogen

Updating the immunogen in a booster vaccine appears to (on average) improve neutralisation titres by 40% to a future variant. However, it is not clear what this means for protection from disease. We have previously established a quantitative relationship between neutralising antibody titres and protection from symptomatic and severe COVID-19^39,40^. We can use this relationship to estimate the additional odds of protection against symptomatic or severe infection that would result from boosting with an updated immunogen, compared to an old booster immunogen.

If an updated booster provides on average a 40% boost in titre compared to an older immunogen, then the odds ratio of protection from severe COVID-19 in a homogeneous population is 1.57 [95% CI: 1.38–1.88]; i.e. the odds of protection from severe disease are increased by 57% compared to boosting with the old antigen. We can also consider how this might play out if we never updated immunogens. For example, if the benefit of updating is constant over different generations of booster antigen, as appears to be the case from Fig. 2 (i.e. a 40% increment for each update), then if an older booster vaccine is two immunogens behind (rather than only one), the odds ratio for protection from severe disease in a population receiving the current booster, compared to one receiving a booster that is two immunogens behind, is predicted to be 1.57^2^ = 2.46 [95% CI = 1.90–3.53].

How this translates into overall protection in a population is dependent on both the background level of immunity in the population and the protection that an old booster would provide^38^. We must therefore consider the predicted advantage of deploying an updated booster over and above the protection that would be achieved using an older booster vaccine. This is shown for a range of values in Supplementary Fig. 4. If an older booster vaccine gave rise to 60% protection in the population against symptomatic disease and 91% protection against severe disease arising from the circulating variant (compared to a naïve population, grey dashed line in Supplementary Fig. 4), then the updated booster would be predicted to increase protection to 68% against symptomatic and 94% against severe disease. If a clinical trial were to be run comparing the old and updated boosters head-to-head, we would expect to observe a relative efficacy of 19% and 30% for the updated immunogen compared to the historical immunogen against symptomatic and severe disease, respectively.

More generally, if we assume that an older booster offers between 30% and 80% protection against symptomatic disease arising from the circulating variant (compared to a naïve population, grey shaded area in Supplementary Fig. 4), and an updated booster immunogen gives a 1.4-fold improvement in neutralisation titres over this older vaccine, we would expect to observe an 11–25% effectiveness of the updated booster vaccine against symptomatic disease and a 23–33% effectiveness against severe disease compared to the older vaccine booster.

## Discussion

The rapid evolution of SARS-CoV-2 variants over time means that decisions on updating booster immunogens must always be made with imperfect information. In particular, decisions on which variant antigen to incorporate into a booster vaccine must be made when the future circulating variant (at the time the vaccine will be deployed) is unknown. Here, we have assembled the (limited) data currently available from past examples to inform this decision. We find that the available evidence on previous booster comparisons suggests that updating vaccine immunogens should provide an average of around a 40% greater boost to neutralisation titres against subsequent season’s SARS-CoV-2 variant, compared to a vaccine containing an immunogen with an older spike variant.

Interestingly, these results suggest that even if ‘updated’ vaccines are not rolled out quickly enough to immunise a population against the currently circulating variant, there remains an immunological benefit to boosting with a new, and possibly divergent antigen. Understanding the mechanisms by which booster immunisation can drive greater neutralising antibody breadth will therefore be key to developing recommendations around the optimal development and deployment of such vaccines in the future.

Several studies have compared clinical protection from COVID-19 following different booster regimes. A major challenge in these studies has often been the use of different booster regimens during specific calendar time intervals, rather than in a (randomised) head-to-head comparison of two regimes. However, studies suggest that BA.1 is around 10% more protective than an ancestral booster^41^ at protecting against symptomatic COVID-19, and up to 50% more effective at preventing severe disease^42,43^. Effectiveness of the BA.4/5 booster against symptomatic disease has been shown to have similar benefits compared to an ancestral booster (additional protection of BA.5 vaccine compared to an Ancestral vaccine was estimated at 8%, [95% CI 0-16%])^44^. In a comparison between boosters containing the BA.1 and BA.5 immunogens, little difference was discernible^45^. Thus, although there are fewer comparisons available, the data on clinical protection support the idea that updated boosters may provide a small increase in clinical protection; however, better prospective data collection on the current level of population immunity, as well as the effectiveness of different booster regiments would add valuable information.

It is important to note that in the four scenarios we have studied to date, this trend has been consistent. Across the comparisons analysed, the estimated benefit of the updated booster immunogen ranged from 1.1- to 1.7-fold (Table 1). However, in future, the benefit of an updated booster immunogen is likely to depend on the antigenic distance between the vaccine immunogen and the variant that eventually circulates. It is possible that to date, by chance, or due to the selection pressure on the virus, an improvement has been observed when using an updated immunogen because the variants that have emerged are progressively more distant from the originating variants^46,47^. It is possible that exceptions or variations to this pattern will arise as the antigenic diversity of the virus continues to increase. Therefore, future studies should assess whether the relative benefit of updating a booster immunogen is related to either the breadth or the magnitude of the protection conferred and whether they can be predicted based on the antigenic relationships between the vaccine immunogen and the subsequent circulating variants.

This analysis has a number of important caveats. Firstly, we have only analysed in vitro neutralisation titres to variant immunogens, and not directly analysed the clinical protection provided by different booster regimens. However, despite this, the estimated increased odds of protection based on this analysis are in broad agreement with the studies outlined above.

Secondly, there were a limited number of studies^10,13,15,16,24,27,29–32^ that provided a head-to-head comparison of in vitro neutralisation titres elicited by different booster immunogens (Table 1). In a subset analysis, using only data from cohorts which included pre- and post-boost neutralisation titres for a future variant after boosting with both an old and updated immunogen (i.e. paired comparison in the same study), we found very similar results (update advantage of 1.37-fold [95% CI: 1.03–1.82] Supplementary Table 7) despite the fact that this is based on less than half the data that was used in the full model. Thirdly, multiple different assays were used across the different studies to measure immunogenicity. We have accounted for this within our model by including an effect for the study from which the data was derived (a proxy for the assay used and the conditions under which it was run). However, the lack of a standardised in vitro neutralisation assay remains a significant impediment to the ongoing comparisons of immunity following vaccination and infection^48,49^.

In addition, the data we have analysed comes from studies in which different variants were assessed at varying time points during the COVID-19 pandemic and after subjects had been exposed to different variants through natural infection. We attempted to account for these differences in our model by incorporating parameters to account for inter-study variation and prior exposure history, but we cannot rule out an additional unexpected impact of exposure to antigenically diverse immunogens that we did not fully capture.

We might speculate how the ongoing circulation of and infection by antigenically distinct variants could affect boosting with different immunogens. On the one hand, a recent infection or a high number of previous infections could decrease the rise in titres conferred by booster vaccination. This might occur if there is a ‘maximum level of boosting’, and the subject is near that maximum before vaccination. Alternatively, recent exposure to an immunogen similar to the one contained in the booster may also affect the level of boosting from the updated immunogen. The impact of recent antigenic exposure, as well as of multiple exposures was not something it was feasible to evaluate with the available data, as most studies did not report on the timing or variant with which subjects had previously been infected, nor on their number of prior infections. However, one study^12^ did report post-boost neutralisation titres following either pre-omicron (cohorts iii and vi) or omicron (cohorts iv and viii) infection. In both cases, neutralisation titres were higher when subjects had been boosted with an updated rather than an older immunogen. Previous studies of the effects of prior infection and boosting in influenza have identified complex interactions^50^, and similar studies should be considered to understand the effects of infection on boosting in COVID-19.

Publication bias also presents a potential challenge to the interpretation of the results of booster comparison studies. For example, 9 studies^4,5,15–19,30,36^ that we identified were sponsored by the companies producing the vaccines. We therefore performed an analysis on a subset of studies^10,13,14,20,21,24,27,29,32^ that reported both pre- and post-boost neutralisation titres and had no pharmaceutical company involvement and found a similar benefit of updating the booster immunogen to that described above (update advantage of 1.39-fold [95% CI: 1.04–1.84], Supplementary Table 8). Additionally, in our multiple regression analysis when we included pharmaceutical involvement as a potential factor, we found this variable was not significant. Both these sensitivity analyses indicate that pharmaceutical sponsorship of studies is unlikely to have influenced our results. However, future studies should aim to provide unbiased analyses of neutralisation titres, preferably using standardised neutralisation assays, and directly comparing alternative booster regimes^51,52^.

Another major limitation of any retrospective study of SARS-CoV-2 variants and immunogenicity is the relatively short history of available data and the inherently unpredictable nature of future antigenic variation. Therefore, the predictions about the relative performance of an updated, compared to an older, immunogen can only be made based on the fairly limited number of prior immunogens that have been tested. The current “omicron” period of SARS-CoV-2 evolution seems characterised by the turnover of mutants displaying antigenic variation but with similar severity, however, alterations to disease severity may emerge^53,54^. In addition, different variants have been co-circulating^55^, suggesting that no variant has a sufficient selection advantage to displace all the others.

Finally, there is no guarantee that a more antigenically escaped, more virulent, or more transmissible variant, will not arise in the future and alter the relative advantage of immunogen updates that we have identified. However, the findings here offer the best predictive tool to date that can be validated against future waves of variants and vaccine updates. Decisions on booster regimes will continue to be made based on the currently available evidence. In this context, we have shown that updating the booster immunogen has, to date, increased neutralisation titres to subsequent circulating variants, and is predicted to have provided a modest increase in protection from COVID-19.

## Methods

### Data acquisition

We searched the VIEW-hub ongoing systematic review database for papers reporting neutralising antibody titres against COVID-19 after vaccination with a COVID-19 variant-immunogen^56^ and indexed prior to 15 August 2023. This search identified 399 studies for screening. We also identified 6 presentations made to the FDA and one paper that was related to one of the papers identified through VIEW-hub, and screened those as well, leading to the screening of 406 papers or presentations.

We screened for studies that were annotated as either (i) including vaccines that contained a BA.1, BA.5, XBB or bivalent immunogen, or (ii) reporting neutralisation titres against the XBB variant. This second screening criterion ensured that we captured all later studies where non-ancestral vaccine immunogens may have been used but where the study may not have been correctly annotated by VIEW-hub. We removed 354 studies from further screening as they did not meet the criteria outlined above.

For studies to be included in our analysis, they must have reported neutralisation titres after boosting, with at least one variant containing immunogen. Studies were excluded if they (i) did not report data after boosting with a variant immunogen, (ii) reported data included in another publication, (iii) did not report neutralisation titres against a variant that occurred chronologically after the vaccine immunogen, (iv) did not specify which immunogen was included in the booster dose, or (v) did not include data from human subjects.

**Fig. 3:**
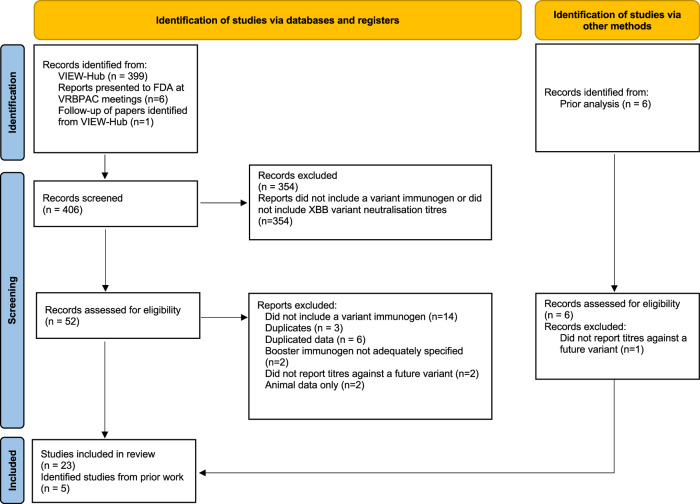
PRISMA flow chart showing studies selected for inclusion in the meta-analysis.

After the screening, we identified a total of 23 of the remaining 52 publications that met our inclusion criteria (left side of Fig. 3). We also included data from a further five studies (right side of Fig. 3), that had previously been identified by us to include neutralisation data after boosting with variant modified vaccines in an earlier analysis^38^ and remained relevant for this analysis. Three of these additional studies included a beta containing vaccine^16,29,30^, which was not indexed in View-Hub, and another two were either omitted^20^, or only had the preprint version indexed in View-Hub^21^ (and the published version was used in our analysis). Together this led to a total of 28 studies from which we could extract data relevant to this analysis(Fig. 3). Full details of the studies identified are given in Supplementary Table 1.

### Data extraction

We extracted neutralisation titres from the identified papers for cohorts:

1. That contained in vitro measurements against ancestral, BA.1, BA.5 and XBB variants.
2. That were unique (i.e. cohorts not already extracted from another paper).
3. Where studies reported outcomes disaggregated with respect to the status of prior infection (i.e. outcomes disaggregated into naïve and previously infected) prior to booster administration, we only extract this disaggregated data.
4. Where studies did not disaggregate data with respect to the status of prior infection, or where data was only disaggregated for either the naïve or previously infected portion of the cohort and was also presented for a mixed cohort. In this case, we extracted the neutralisation titres for the mixed cohort.
5. For which the most recent “boost” was by vaccination (not infection).
6. With subjects who had been given at least two prior doses of a vaccine.

We extracted neutralisation titres for the Ancestral, BA.1, BA.5 and XBB variants. Where a bivalent vaccine contained multiple immunogens (e.g. Ancestral + BA.5 or early VoC + BA.1) we classified the vaccine immunogen to be that of the latest chronological variant.

### Mixed effects model

We constructed a mixed effects model to determine if the neutralising antibody response following boosting (either the fold rise in neutralising antibodies or the absolute neutralising antibody titres following boosting) was dependent on the immunogen contained within the booster.

The model included a fixed effect for the booster immunogen (old or updated) and accounted for the study from which the data came by incorporating a random effect for the intercept with a grouping structure on the study from which the data came. It also accounted for the number of subjects within each cohort by weighting the contribution of each data point so that more weight was given to larger cohorts.

We also constructed an extended model that included the effects outlined above and additionally included a fixed effect for the exposure history of a cohort (average number of prior exposures within a cohort) and an additional random effect on the intercept with a grouping structure on the pairing of the data within a study (so that cohorts from the same study with the same exposure history, but with different booster immunogens, could be paired together within a study). The extended model also accounted for the comparison that was being made (i.e. comparison number 1, 2, 3 or 4 from Fig. 1) as a grouping structure on the random effect for both the intercept and the booster immunogen. Full details of the model are provided in the supplementary materials.

### Predicting the clinical benefit

We have previously established a relationship between neutralising antibody titres and protection from symptomatic and severe COVID-19^39^ based on a logistic relationship between neutralisation titres and protection from disease. This relationship is described by

1 $${{{\rm{P}}}}\left(n\right)=\frac{1}{1-{e}^{-k\left(n-{n}_{50}\right)}}$$

where ${{{\rm{P}}}}\left(n\right)$ is the protection conferred (compared to a naive individual) for a neutralisation titre of $n$ (with $n$ is measured on a ${\log }_{10}$ scale) and ${n}_{50}$ is the neutralisation titre at which an individual will be half as likely to acquire disease as a naive individual. We have previously estimated the value of $k$ as $k$ = 3.10 (95% CI: 2.19–4.38) for symptomatic disease and $k$ = 3.08 (95% CI: 2.19–4.32) for severe disease.

### Determining the odds ratio of protection

For a logistic function, such as the one described in Eq. (1) above, the odds of protection for a neutralisation titre of $n$ are defined as

2 $${{{\rm{ODDs}}}}\left(n\right)={e}^{k\left(n-{n}_{50}\right)}$$

We denote the odds ratio of protection for an individual with a $\beta$-fold increase in neutralisation titres by ${{{\rm{OR}}}}\left({{{\rm{\beta }}}}\right)$. We can determine ${{{\rm{OR}}}}\left({{{\rm{\beta }}}}\right)$ using Eq. (2) as the ratio of the odds of protection for an individual with a $\beta$-fold increase in titres (or log_10_ β increase on a log_10_ scale), compared to the odds of protection for an individual with their baseline neutralisation titres. We denote the baseline neutralisation titres by ${n}_{0}$ (where again ${n}_{0}$ is measured on a ${\log }_{10}$ scale). We can derive ${{{\rm{OR}}}}\left({{{\rm{\beta }}}}\right)$ as:

3 $${{{\rm{OR}}}}\left({{{\rm{\beta }}}}\right)=\frac{{{{\rm{ODDs}}}}\left({\log }_{10}{{{\rm{\beta }}}}+{n}_{0}\right)}{{{{\rm{ODDs}}}}\left({n}_{0}\right)}={e}^{k{\log }_{10}{{{\rm{\beta }}}}}$$

### Determining the vaccine effectiveness

The translation of an improvement in neutralisation titres to the overall protection in a population, given (i) a certain level of background level of immunity and (ii) the protection that an old booster would provide is performed in a similar manner to that described in ref. ^38^.

### Ethics statement

This work was approved under the UNSW Sydney Human Research Ethics Committee (approval HC200242).

### Reporting summary

Further information on research design is available in the Nature Portfolio Reporting Summary linked to this article.

## Supplementary information

Supplementary Information

Peer Review File

Reporting Summary

## Data Availability

Data is available at: https://github.com/iap-sydney/Prediciting-Benefit-of-Vaccine-Updates10.5281/zenodo.13131679.

## Code availability

Code is available at: https://github.com/iap-sydney/Prediciting-Benefit-of-Vaccine-Updates10.5281/zenodo.13131679.
