# Supplementary material for: Predicting COVID-19 booster immunogenicity against future SARS-CoV-2 variants and the benefits of vaccine updates
Source: Nat Commun. 2024 Sep 27;15:8395. doi: 10.1038/s41467-024-52194-9 (PMC11436652; doi:10.1038/s41467-024-52194-9)
Supplement: Supplementary file 1 — Supplementary Information [file 41467_2024_52194_MOESM1_ESM.pdf]

Supplementary Materials

Mixed Effects Model

As outlined in the main manuscript, the aim of our analysis was to consider pairs of potential immunogens, one designated as ‘old’ and one as ‘updated’, and compare their immunogenicity against a future variant, as outlined in Figure 1 of the main manuscript.

We constructed a model that modelled either the fold rise in neutralising antibody titres or the absolute value of the neutralising antibody titres after boosting. The model included fixed effects for the booster immunogen (old or updated) and incorporated a random effect on the intercept with a grouping structure of the study from which the data came. The model assumes a linear relationship between neutralisation capabilities and the neutralisation titre reported by the assay. The contribution of each data point was weighted by the square root of the number of subjects in the cohort from which it was derived, thereby giving more weight to larger cohorts.

The general form of the model of the antibody titres for cohort i, T_i is given by:

$$\log_{10}(T_i) = c_1 + c_2 I_i + s_i \qquad \text{Equation S1}$$

where

- $c_1$is a constant representing the fixed effect for the intercept
- c_2 is a constant representing the fixed effect for the difference between old and updated immunogens in the log10 of the fold rise in neutralising antibody titre
- I_i is a dummy variable determining whether the booster immunogen given to cohort i was old (I_i = old) or updated (I_i = updated).
- s_i is a random effect with a grouping structure of the study from which the data came.

We also constructed an extended model that accounted for the different exposure histories of subjects within the different cohorts. In this extended model, cohorts were paired if they were from the same study and had the same exposure history, but were given different booster immunogens. In addition to the effects described in the model in Equation S1, this extended model included a fixed effect for the exposure history of a cohort (average number of prior exposures within a cohort), a random effect on the intercept with a grouping structure on the pairing of the cohorts within a study (so that cohorts from the same study with the same exposure history, but with different booster immunogens, could be paired together within a study) and random effects on both the intercept and the contribution of the booster immunogen with grouping structures on the comparison that was being made (i.e. comparison number 1, 2, 3 or 4 from Figure

1). The general form of the extended model for the antibody titres after boosting for cohort i, T_i is given by:

$$\log_{10}(T_i) = c_1 + c_2 I_i + c_3 E_i + s_i + p_i + c_{1i} + c_{2i} I_i \qquad \text{Equation S2}$$

where

- c_1 is a constant representing the fixed effect for the intercept
- c_2 is a constant representing the fixed effect for the difference between old and updated immunogens in the log10 of the fold rise in neutralising antibody titre
- c_3 is a constant representing the fixed effect for each additional exposure to the virus
- I_i is a dummy variable determining whether the booster immunogen given to cohort i was old (I_i = old) or updated (I_i = updated).
- E_i is a continuous variable representing the average number of prior exposures for the subjects within cohort i.
- s_i is a study-specific random effect.
- p_i is a pairing-specific random effect.
- c_{1i} and c_{2i} are comparison-specific random effects (i.e., comparisons 1-4), with c_{1i} being a random effect on the intercept and c_{2i} being a random effect on the immunogen type.

We defined the number of exposures for cohort i, E_i, as the sum of (i) the average of the number of prior vaccine doses received within the cohort (e.g. individuals receiving 2, 3 or 4 vaccine doses) and (ii) their prior infection status. Prior infection status was defined as 0 (uninfected) or 1 (infected). In cohorts with mixed infection status, the prior infection status of the cohort was defined to be the proportion of the cohort that had undergone prior infection. If such information was not reported, the prior infection status of the cohort was set as 0.5.

Statistical and model fitting

All analyses were conducted in R (v4.3.0) using library glmmTMB (v1.1.7). All reported p-values are based on the significance of the estimated parameters (Wald test), and the 95% CI are calculated directly from the estimated standard error of the parameters.

Impact of boosting after multiple exposures to the virus – empirical data

A major challenge in comparing different booster regimens is that they were tested at different stages of the pandemic. For example, testing of the early VoC boosters generally occurred in subjects that had received only a primary vaccination course or had received a single booster dose. By contrast, comparison of the BA.1 and BA.5 bivalent booster vaccines occurred in a population that had up to 4 prior exposures (infections or vaccinations).

To determine the impact of prior exposures we initially grouped the extracted data by the number of prior exposures a cohort has previously experienced (prior to their boost). Where a cohort contained a mix of infected and uninfected subjects, and so had a non-integer mean number of exposures, we rounded the number of exposures to the nearest integer for this grouped analysis.

We observed a significant decrease in the magnitude of booster effect (for all variants) with increasing number of prior exposures, however, the estimated benefit of the updated immunogen remained constant across the number of prior exposures (Supplementary Figure 2). We note that only that the *benefit* of a vaccine boost is reduced for subsequent exposures, not the absolute neutralisation titres. For each variant, considering the boosting of cohorts that have had primary vaccination and up to two additional exposures, each immunogen exposure resulted in higher post-boost neutralisation titres (Supplementary Figure 3).

Impact of boosting after multiple exposures to the virus – results of modelling

We next determined the contribution of exposure history to the rise in neutralisation titres following boosting from our mixed effects model. In this case we treated the number of prior exposures as a continuous variable. After accounting for different vaccine immunogens and comparisons, the number of prior exposures to a SARS-CoV-2 antigen still had a significant impact on the rise in neutralisation titres after boosting ($p<0.001$). For each subsequent exposure to a SARS-CoV-2 immunogen (up to the third exposure either from vaccination or infection). The magnitude of boosting decreased, on average, by 2.5-fold [95% CI 2.1-3.1] (negative slope of fitted model in Supplementary Figure 1B).

We observe that if this decrease were to continue, this would imply a fold rise of less than one, when boosting cohorts who had received four or more additional exposures after their primary series vaccination (i.e. a negative effect of boosting). Obviously, this is not biologically reasonable, and therefore this model is only applicable for the range of exposures over which we have available data with which to parameterise it. It is highly likely that, rather than having a fixed decrease of 2.5-fold in the magnitude of boosting for each exposure, this decrease may be smaller for each subsequent exposure. However, determining this will only be possible as more data becomes available for analysis.

Supplementary Figures

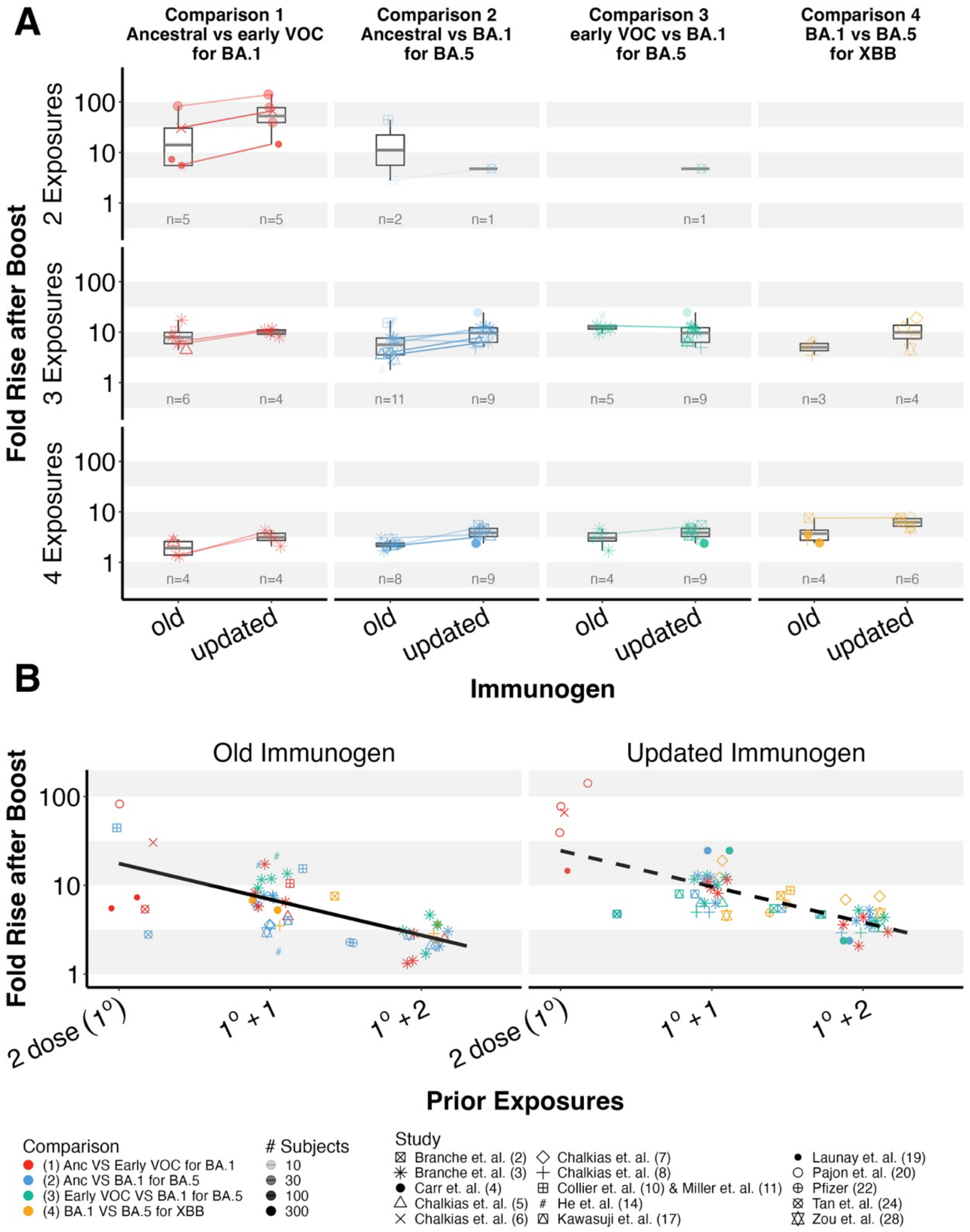

Supplementary Figure 1

A) Representation of the data used to inform the mixed effects model of the fold rise in neutralisation titres after boosting with different immunogens. Data is stratified by the number of prior exposures (rows) and the comparison number (columns). Lines connect paired cohorts from the same study. (B) Model fits (lines) to data (symbols) showing the fold rise in neutralisation titres after boosting with an old (left) or updated (right) immunogen. Colours of symbols correspond to the different comparisons outlined in Figure 2 of the main paper.

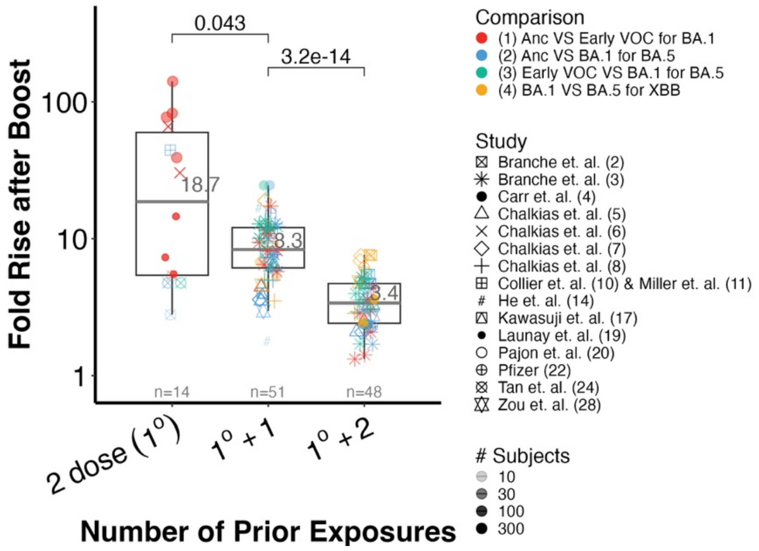

Supplementary Figure 2

Fold rises in neutralisation titres after boosting with either an old or updated immunogen, stratified by the number of prior exposures within each cohort. Data are coloured by the comparison number to which they relate, and colours correspond to the comparisons depicted in Figure 2 of the main paper. Grey lines in the middle of the box plots (and numbers) indicate mean values (not median values). Comparison values across the top are p-values from two-sided unpaired t-tests.

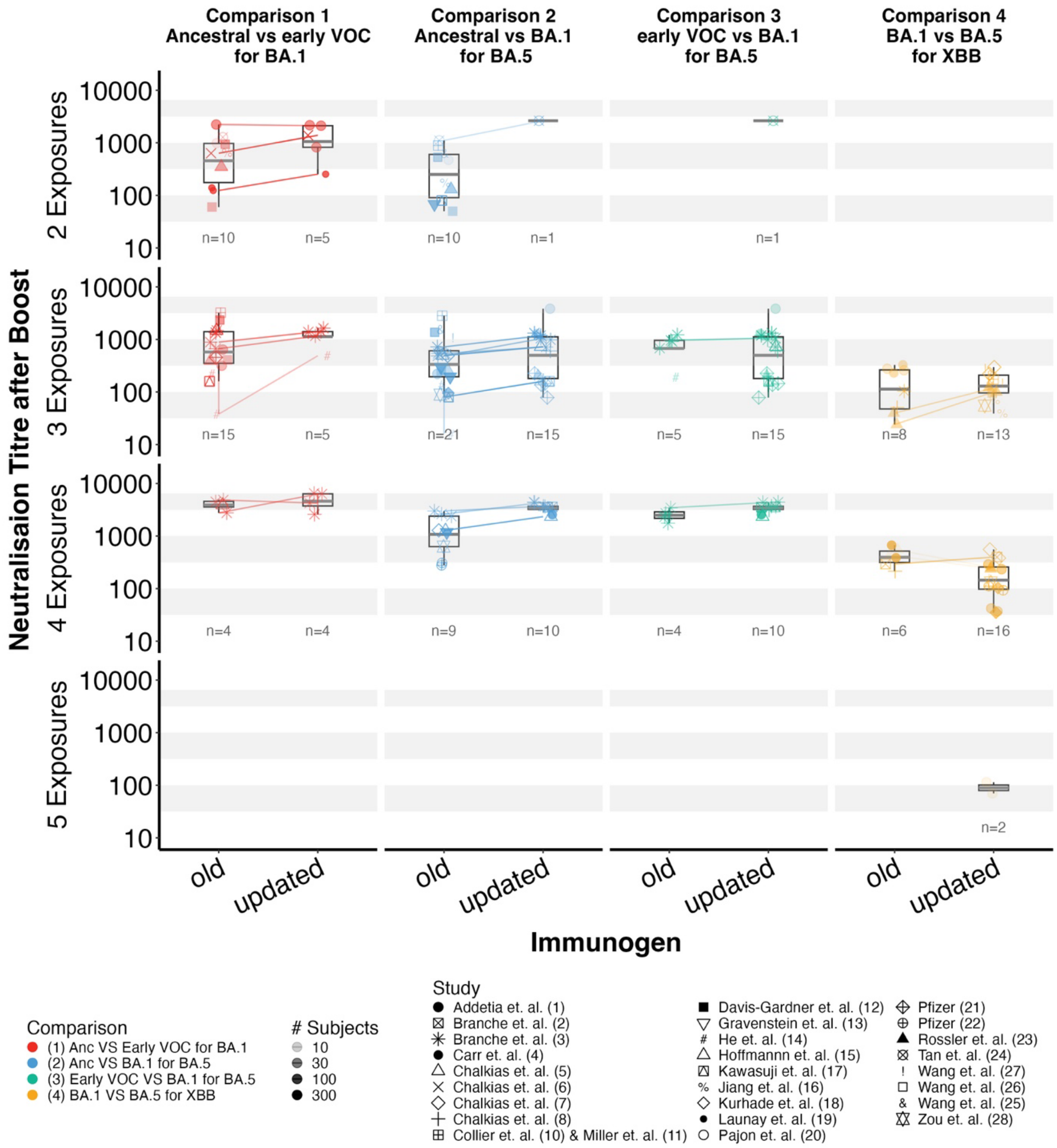

Supplementary Figure 3

Representation of the data used to inform the mixed effects model of the absolute neutralisation titres after boosting with either an old or an updated immunogen. Data is stratified by the number of prior exposures (rows) and the comparison number (columns). Colours of symbols correspond to the different comparisons outlined in Figure 2 of the main paper. Lines connect paired cohorts from the same study.

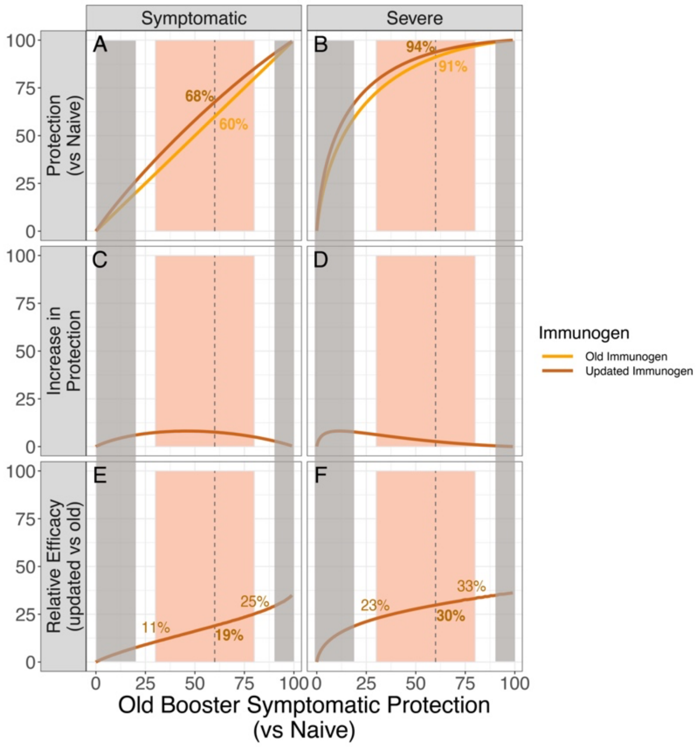

Supplementary Figure 4

Impact of a vaccine containing the updated immunogen that confers a 40% increase in neutralising antibody titres on protection from symptomatic (A,C,E) and severe disease (B,D,F). The x-axis shows varying levels of protection against symptomatic disease arising from the circulating variant that would be conferred by using the older immunogen (as compared to a naïve individual). Panels A and B show absolute efficacy of the old and updated boosters (compared to a naïve population). Panels C and D show the absolute improvement in efficacy (for an updated immunogen compared to an older immunogen) and Panels E and F show the relative efficacy that would be obtained in a clinical trial directly comparing the old and updated immunogens. Grey dashed line shows the 60% protection level example discussed in the manuscript. Red shading shows the 30-80% range of old booster protection discussed in the manuscript. Numbers on the figure show percentages referenced in the text. We note that "old booster protection" of under 20%- representing an ineffectual boost and greater than 90% - representing a boost that is as effective as the original vaccines against a non-escaped variant are not realistic. These areas are shaded in grey and should be interpreted with caution.

Supplementary Tables

Reference (Ref in main paper)	Conflict	Has pre-boost data?	Immunogen	Relevant Variants	Number of Prior Doses	Prior Infectious Status	Relevant Data
[1] (12)	None, however some authors are employees of Vir Biotechnology.	No	Ancestral, BA.1 bivalent, BA.5 bivalent	Ancestral, BA.1, BA.5, XBB	Multiple cohorts with different numbers of prior doses.<br>*(i) Wu4* - 3 doses<br>*(ii) Wu/BA.5 biv* - mean 3.5 doses (range 2-4)<br>*(iii) Pre-Omicron -Wu/BA.5 biv* - mean 3.6 doses (range 3-4)<br>*(iv) Omicron BT -Wu/BA.5 biv* - mean 3.9 doses (range 3-5)<br>*(v) Wu3 mono* - 2 doses<br>*(vi) Pre-Omicron–Wu3 mono* - 2 doses<br>*(vii) Wu/BA.1 biv* - 3 doses	Multiple cohorts with different prior infection statuses.<br>*(i) Wu4* - Uninfected<br>*(ii) Wu/BA.5 biv* - Uninfected<br><br>*(iii) Pre-Omicron–Wu/BA.5 biv* - Infected<br>*(iv) Omicron BT–Wu/BA.5 biv* - Infected<br>*(v) Wu3 mono* - Uninfected<br>*(vi) Pre-Omicron–Wu3 mono* - Infected<br>*(vii) Wu/BA.1 biv* - Uninfected	Figure 4 (A-B), cohorts (i-vii)<br>Data not extracted from cohort (viii) Omicron BT–Wu/BA.1 biv group as it was not clear whether the most recent exposure was an infection or a vaccination.
[2] (13)	None	Yes	BA.1 bivalent, BA.5 bivalent	Ancestral, BA.1, BA.5, XBB	3 doses	Mixed<br>*Monogram group -*<br>Pfizer BA.1 bivalent group - 79% infected, 21% uninfected.<br>Pfizer BA.4/5 bivalent groups - 75% infected, 25% uninfected.<br>*Duke group -*<br>Mix of infected and uninfected, exact proportion not reported.	Figure 1(A-D), Table S6 and Table S7<br>Data from uninfected cohorts not used as no similar infected cohorts presented.

[3] (10)	None	Yes	Ancestral, early VOC, BA.1 monovalent, BA.1 bivalent	Ancestral, BA.1, BA.5, XBB	3 doses	Uninfected, Infected	Tables S6-S11
[4] (14)	None	Yes	BA.1 bivalent	Ancestral, BA.1, BA.5, XBB	3 doses	Uninfected, Infected	Figure<br>Only data from uninfected and infected cohorts.
[5] (15)	Supported by Moderna	Yes	Ancestral, BA.1 bivalent	Ancestral, BA.1, BA.5	3 doses	Uninfected, Infected	Figure 3, Figure S3, Figure S4<br>Only data from uninfected and infected cohorts
[6] (16)	Supported by Moderna	Yes	Ancestral, early VOC	Ancestral, BA.1	2 doses	Uninfected<br>(up to 4% of a cohort has experienced prior infection, but these were still classified as uninfected)	Table S3
[7] (17)	Supported by Moderna	Yes	Ancestral, BA.5 bivalent	Ancestral, BA.5, XBB	3 doses	Uninfected, Infected	Figure 3, Table S11<br>Only data from uninfected and infected cohorts.
[8] (18)	Supported by Moderna	Yes	BA.1 bivalent, BA.5 bivalent	BA.5, XBB	3 doses	Uninfected, Infected	Figure S5, Table S10<br>Only BA.1 variant data was used, as other data from this preprint is included in published form in reference [7].
[9] (19)	Supported by Moderna	Yes	XBB.1.5 monovalent, bivalent vaccine containing BA.5 and XBB.1.5	Ancestral, BA.5, XBB	4 doses	Uninfected, Infected	Figure 1 and Figure 2<br>Only data from uninfected and infected cohorts. Data only included in Figure 3 and not in the main analysis as no titres were provided after immunising with one of the relevant immunogens.

[10, 11] (20,21)	None	Yes	Ancestral, BA.5 bivalent	Ancestral, BA.1, BA.5, XBB	*Ancestral booster, uninfected cohort* - 2 doses<br>*Ancestral booster, mixed prior infection cohort* - mean 2.9 doses (range 2-3).<br>*BA.5 bivalent booster, mixed prior infection cohort* - mean 3.1 doses (range 2-4).	Uninfected[11]<br>Mixed (33% infected, 67% uninfected) [10, 11].	Figure 1[10] and Figure S1[10]<br>Figure 1(B-D)[11]<br>(excluding WT and BA.5 data from Figure 1C and D)
[12] (22)	None	No	Ancestral, BA.5 bivalent	Ancestral, BA.1, BA.5, XBB	2 doses, 3 doses	Uninfected and Mixed For mixed cohorts:<br>*Ancestral booster cohort* - 27% infected, 73% uninfected.<br>*BA.5 booster cohort* - 17% infected, 83% uninfected.	Figure 1 (A - C)
[13] (23)	None	No	Ancestral, BA.5 bivalent	Ancestral, BA.5	2 doses, 3 doses, 4 doses	Uninfected, Infected	Table 2
[14] (24)	None	Yes	Ancestral, early VOC, BA.5 bivalent	Ancestral, BA.1, BA.5	3 doses	Uninfected	Figure 2, Figure 3
[15] (25)	None	No	Ancestral, BA.5 bivalent	Ancestral, BA.1, BA.5	2 doses, 3 doses	Uninfected, Infected	Figure A, Figure B
[16] (26)	None	No	Ancestral, BA.5 bivalent	Ancestral, BA.1, BA.5, XBB	2 doses, 3 doses	Uninfected	Figure 3
[17] (27)	None	Yes, however pooled across all cohorts	Ancestral, BA.1 bivalent	Ancestral, BA.1, BA.5	2 doses, 3 doses	Uninfected	Figure 3B, Figure S2

[18] (28)	No, however one author received compensation from Pfizer for COVID-19 vaccine development.	No	Ancestral, BA.5 bivalent	Ancestral, BA.5, XBB	Multiple cohorts: *Ancestral booster* - 3 doses *BA.5 booster, uninfected* - mean 3.6 doses (range 2-4). *BA.5 booster, infected* - mean 3.1 doses (range 2-4).	Uninfected, Infected	Figure 1
[19] (29)	No, however two authors are consultants to multiple vaccine companies and third is a consultant to Pfizer only.	Yes	Ancestral, early VOC	Ancestral, BA.1	2 doses	Uninfected	Figure 1
[20] (30)	Authors work for Moderna	Yes	Ancestral, early VOC	Ancestral, BA.1	2 doses	Uninfected	Figure S6
[21] (4)	Pfizer presentation	Yes	Ancestral, BA.1 monovalent , BA.1 bivalent	Ancestral, BA.1, BA.5	2 doses, 3 doses	Uninfected	Slides labelled CC8-CC19
[22] (5)	Pfizer presentation	Yes	Ancestral, BA.5 bivalent	BA.5, XBB	3 doses	Mixed	Slide 9

[23] (31)	None. One author holds stocks in Pfizer.	No	Ancestral, BA.1 bivalent, BA.5 bivalent	Ancestral, BA.1, BA.5, XBB	2 doses, 3 doses	Uninfected, infected	Figure 1, Figure 3
[24] (32)	None	Yes	Ancestral, BA.1 bivalent	Ancestral, BA.1, BA.5	2 doses, 1 dose	Mixed<br>*Cohort receiving Pfizer ancestral booster* - 11% infected, 89% uninfected.<br>*Cohort receiving Moderna BA.1 bivalent booster* - 32% infected, 68% uninfected.	Figure 3<br>Data from cohorts with only one prior vaccination was not included.
[25] (33)	No, however one author is on the scientific advisory board of Janssen Biotech. and another has relationships with a number of medical development companies.	No	Ancestral, BA.5 bivalent	Ancestral, BA.5, XBB	2 doses, 3 doses	Uninfected	Figure 2

[26] (34)	No, however one author is on the scientific advisory board of Janssen Biotech. and another has relationships with a number of medical development companies.	No	Ancestral, BA.5 bivalent	Ancestral, BA.1, BA.5	2 doses, 3 doses	Uninfected	Figure 1
[27] (35)	None	No	Ancestral, BA.5 bivalent	Ancestral, BA.5, XBB	3 doses, 4 doses	Uninfected	Figure 1
[28] (36)	Supported by Pfizer	Yes	Ancestral, BA.5 bivalent	Ancestral, BA.5, XBB	3 doses	Uninfected, Infected	Figure 1, Figure S1, Table S4 Only data from uninfected and infected cohorts.

Supplementary Table 1: Studies from which data were extracted to use in this analysis.

**Comparison Number**	**Old Immunogen**	**Updated Immunogen**	**Future Variant**	**Number of contributing studies**
**Comparison 1 (Red)**	Ancestral	Early VoC	BA.1	15
**Comparison 2 (Blue)**	Ancestral	BA.1	BA.5	24
**Comparison 3 (Green)**	Early VoC	BA.1	BA.5	11
**Comparison 4 (Orange)**	BA.1	BA.5	XBB	15

Supplementary Table 2: Description of the comparisons made between different vaccine immunogens to induce neutralisation against future variants, and the number of studies with data that could contribute to each comparison.

Sensitivity Analyses

Supplementary tables 3 – 8 include the parameter values from mixed effects models that were run as part of the sensitivity analyses for the model. Values in the tables are determined using the glmmTMB function from the R package glmmTMB, in which p-values are obtained from a two-sided Wald test. in Values are given to 3 significant figures. P-values less than 1e-5 are reported as <1e-5.

**Dependant Variable**	**Parameter**	**Value**	**Std err**	**P-value**
Neutralisation titres after boosting	Intercept	1.43	0.151	<0.0001
	Immunogen (updated vs older)	0.189	0.0712	7.82e-3
	Exposure number	-0.661	0.169	<0.0001

Supplementary Table 3
Parameter values for the mixed effects model described in Equation S2, that predicts the fold-rise in neutralisation titres following boosting and is fit only to the data shown in Supplementary Figure 1A from cohorts without prior infection.

Dependant Variable	Parameter	Value	Std err	P-value
Neutralisation titres after boosting	Intercept	1.29	0.0856	<0.0001
	Immunogen (updated vs older)	0.198	0.0654	2.47e-3
	Exposure number	-0.438	0.0470	<0.0001

Supplementary Table 4
Parameter values for the mixed effects model described in Equation S2, that predicts the fold-rise in neutralisation titres following boosting and is fit only to the data shown in Supplementary Figure 1A from cohorts with a homogeneous prior infection status.

Dependant Variable	Parameter	Value	Std err	P-value
Neutralisation titres after boosting	Intercept	1.10	0.117	<0.0001
	Immunogen (updated vs older)	0.152	0.0625	0.0147
	Exposure number	-0.330	0.0562	<0.0001

Supplementary Table 5
Parameter values for the mixed effects model described in Equation S2, that predicts the fold-rise in neutralisation titres following boosting and is fit only to the data shown in Supplementary Figure 1A that did not include a comparison with a vaccine that contained an immunogen from an early VoC, i.e. based only on data from comparisons 2 and 4 from Supplementary Figure 1A

Dependant Variable	Parameter	Value	Std err	P-value
Neutralisation titres after boosting	Intercept	2.22	0.234	<0.0001
	Immunogen (updated vs older)	0.171	0.0412	<0.0001
	Exposure number	0.328	0.0449	<0.0001

Supplementary Table 6
Parameter values for the mixed effects model described in Equation S2, that predicts the absolute neutralisation titres following boosting and is fit to the data shown in Supplementary Figure 3.

Dependant Variable	Parameter	Value	Std err	P-value
Neutralisation titres after boosting	Intercept	1.27	0.176	<0.0001
	Immunogen (updated vs older)	0.136	0.0652	0.0371
	Exposure number	-0.417	0.0411	<0.0001

Supplementary Table 7
Parameter values for the mixed effects model described in Equation S2, that predicts the fold-rise in neutralisation titres following boosting and is fit only to the paired data shown in Supplementary Figure 1A.

Dependant Variable	Parameter	Value	Std err	P-value
Neutralisation titres after boosting	Intercept	1.29	0.132	<0.0001
	Immunogen (updated vs older)	0.142	0.0662	0.0314
	Exposure number	-0.495	0.0560	<0.0001

Supplementary Table 8
Parameter values for the mixed effects model described in Equation S2, that predicts the fold-rise in neutralisation titres following boosting and is fit only to the data shown in Supplementary Figure 1A that did not have pharmaceutical sponsorship.

References

1. Addetia A*, et al.* Neutralization, effector function and immune imprinting of Omicron variants. *Nature* 621, 592-601 (2023).
2. Branche AR*, et al.* Immunogenicity of the BA.1 and BA.4/BA.5 Severe Acute Respiratory Syndrome Coronavirus 2 Bivalent Boosts: Preliminary Results From the COVAIL Randomized Clinical Trial. *Clin Infect Dis* 77, 560-564 (2023).
3. Branche AR*, et al.* Comparison of bivalent and monovalent SARS-CoV-2 variant vaccines: the phase 2 randomized open-label COVAIL trial. *Nat Med* 29, 2334-2346 (2023).
4. Carr EJ*, et al.* Neutralising immunity to omicron sublineages BQ.1.1, XBB, and XBB.1.5 in healthy adults is boosted by bivalent BA.1-containing mRNA vaccination and previous Omicron infection. *Lancet Infect Dis* 23, 781-784 (2023).
5. Chalkias S*, et al.* A Bivalent Omicron-Containing Booster Vaccine against Covid-19. *N Engl J Med* 387, 1279-1291 (2022).
6. Chalkias S*, et al.* Safety, immunogenicity and antibody persistence of a bivalent Beta-containing booster vaccine against COVID-19: a phase 2/3 trial. *Nat Med* 28, 2388-2397 (2022).
7. Chalkias S*, et al.* Original SARS-CoV-2 monovalent and Omicron BA.4/BA.5 bivalent COVID-19 mRNA vaccines: phase 2/3 trial interim results. *Nat Med* 29, 2325-2333 (2023).

8. Chalkias S*, et al.* Safety and Immunogenicity of Omicron BA.4/BA.5 Bivalent Vaccine Against Covid-19. *medRxiv*, (2022).
9. Chalkias S*, et al.* Safety and Immunogenicity of XBB. 1.5-Containing mRNA Vaccines. *medRxiv*, (2023).
10. Collier AY*, et al.* Immunogenicity of BA.5 Bivalent mRNA Vaccine Boosters. *N Engl J Med* 388, 565-567 (2023).
11. Miller J*, et al.* Substantial Neutralization Escape by SARS-CoV-2 Omicron Variants BQ.1.1 and XBB.1. *N Engl J Med* 388, 662-664 (2023).
12. Davis-Gardner ME*, et al.* Neutralization against BA.2.75.2, BQ.1.1, and XBB from mRNA Bivalent Booster. *N Engl J Med* 388, 183-185 (2023).
13. Gravenstein S*, et al.* Durability of immunity and clinical protection in nursing home residents following bivalent SARS-CoV-2 vaccination. *medRxiv*, (2023).
14. He Q*, et al.* The Bivalent COVID-19 Booster Immunization after Three Doses of Inactivated Vaccine Augments the Neutralizing Antibody Response against Circulating Omicron Sublineages. *J Clin Med* 12, (2022).
15. Hoffmann M*, et al.* Effect of hybrid immunity and bivalent booster vaccination on omicron sublineage neutralisation. *Lancet Infect Dis* 23, 25-28 (2023).
16. Jiang N*, et al.* Bivalent mRNA vaccine improves antibody-mediated neutralization of many SARS-CoV-2 Omicron lineage variants. *bioRxiv*, (2023).
17. Kawasuji H*, et al.* Neutralizing Antibody Response of the Wild-Type/Omicron BA.1 Bivalent Vaccine as the Second Booster Dose against Omicron BA.2 and BA.5. *Microbiol Spectr* 11, e0513122 (2023).
18. Kurhade C*, et al.* Low neutralization of SARS-CoV-2 Omicron BA.2.75.2, BQ.1.1 and XBB.1 by parental mRNA vaccine or a BA.5 bivalent booster. *Nat Med* 29, 344-347 (2023).
19. Launay O*, et al.* Immunogenicity and Safety of Beta-Adjuvanted Recombinant Booster Vaccine. *N Engl J Med* 387, 374-376 (2022).
20. Pajon R*, et al.* SARS-CoV-2 Omicron Variant Neutralization after mRNA-1273 Booster Vaccination. *N Engl J Med* 386, 1088-1091 (2022).
21. Pfizer. Vaccines and Related Biological Products Advisory Committee June 28, 2022 Meeting Presentation- Pfizer/BioNTech COVID-19 Omicron-Modified Vaccine Options.) (2022).
22. Pfizer. Vaccines and Related Biological Products Advisory Committee June 15, 2023 Meeting Presentation- Pfizer: 2023-2024 COVID19 Vaccine Formula- Clinical and Preclinical Supportive Data.) (2023).
23. Rossler A*, et al.* Characterizing SARS-CoV-2 neutralization profiles after bivalent boosting using antigenic cartography. *Nat Commun* 14, 5224 (2023).
24. Tan NH*, et al.* Immunogenicity of bivalent omicron (BA.1) booster vaccination after different priming regimens in health-care workers in the Netherlands (SWITCH ON): results from the direct boost group of an open-label, multicentre, randomised controlled trial. *Lancet Infect Dis* 23, 901-913 (2023).

25. Wang Q*, et al.* Alarming antibody evasion properties of rising SARS-CoV-2 BQ and XBB subvariants. *Cell* 186, 279-286.e278 (2023).
26. Wang Q*, et al.* Antibody Response to Omicron BA.4-BA.5 Bivalent Booster. *N Engl J Med* 388, 567-569 (2023).
27. Wang W*, et al.* Bivalent Coronavirus Disease 2019 Vaccine Antibody Responses to Omicron Variants Suggest That Responses to Divergent Variants Would Be Improved With Matched Vaccine Antigens. *J Infect Dis* 228, 439-443 (2023).
28. Zou J*, et al.* Neutralization of BA.4-BA.5, BA.4.6, BA.2.75.2, BQ.1.1, and XBB.1 with Bivalent Vaccine. *N Engl J Med* 388, 854-857 (2023).
