# Supplementary material for: Predicting COVID-19 booster immunogenicity against future SARS-CoV-2 variants and the benefits of vaccine updates
Source: Nat Commun. 2024 Sep 27;15:8395. doi: 10.1038/s41467-024-52194-9 (PMC11436652; doi:10.1038/s41467-024-52194-9)
Supplement: Supplementary file 2 — Peer Review File [file 41467_2024_52194_MOESM2_ESM.pdf]

nature portfolio

Peer Review File

Predicting COVID-19 booster immunogenicity against future SARS-CoV-2 variants and the benefits of vaccine updates

Open Access This file is licensed under a Creative Commons Attribution 4.0 International License, which permits use, sharing, adaptation, distribution and reproduction in any medium or format, as long as you give appropriate credit to the original author(s) and the source, provide a link to the Creative Commons license, and indicate if changes were made. In the cases where the authors are anonymous, such as is the case for the reports of anonymous peer reviewers, author attribution should be to 'Anonymous Referee' followed by a clear attribution to the source work. The images or other third party material in this file are included in the article's Creative Commons license, unless indicated otherwise in a credit line to the material. If material is not included in the article's Creative Commons license and your intended use is not permitted by statutory regulation or exceeds the permitted use, you will need to obtain permission directly from the copyright holder. To view a copy of this license, visit http://creativecommons.org/licenses/by/4.0/.

REVIEWER COMMENTS

Reviewer #1 (Remarks to the Author):

Cromer et al. perform a meta-analysis of published studies to model the benefit of boosting with an updated COVID19-vaccine compared to a previous vaccine antigen on titers of neutralizing antibodies against future variants. They find that the boost in neutralizing antibody titers was on average 40% higher against a future variant when using a more advanced variant as booster compared to an older variant. This study is interesting and important to judge the need for COVID-19 vaccine updates.

• Abstract line 16/17 + Introduction lines 37-39: Decisions on vaccine updates are not only been made based on neutralizing antibody titers but also on estimates of vaccine efficacy..
• Figure 2: Symbols are not explained.
• N should be added to figure legends.
• Lines 164-172: This paragraph is a bit confusing. In Figure 2B not all of the 27 studies could be used as not all studies contained pre-boost titers. It might be helpful to already give here the info that for Figure B only 16 studies were used.
• Information in Table 2 might be difficult to understand for a broader audience. Some more explanation might be helpful.
• Figure 3: Legend seems to be incomplete
• Line 379: "COVID-19 variants" might be changed to "SARS-CoV-2 variants" as the authors talk here about virus variants and not disease. Alternatively, "COVID-19 vaccine variants" may be appropriate.
• Some references seem to be incomplete such as ref. 29, 30, 31 etc.
• Uncertainty in the number of prior infections may be a limitation of the modelling approach as the authors show that the increased benefit of an updated compared to a previous vaccine antigen becomes smaller after multiple exposures. The authors define infection history as 0 (uninfected) or 1 (infected) or the proportion of the cohort that had been infected. Multiple infections and especially the infecting variant and time since infection might have a big influence on fold-increase of neutralizing titers after booster. As many of these factors are unknown for the majority of studies it might be difficult to incorporate them. However, it may be good to add these limitations to the discussion.

Reviewer #2 (Remarks to the Author):

Cromer et al. have performed a systematic literature review and statistical analysis of data on the effect of vaccine updates on boosting neutralizing antibody titers to a future infecting variant of SARS-CoV-2. The study leverages the group's previous work establishing an association between neutralizing antibody titers and protection to then apply the modeling results here to interpretation of the effect of vaccine updates on improving protection against future circulating variants. The manuscript is overall well-written, and many of the sensitivity analyses are justified. There were some instances where I was not able to follow the authors line of logic and have noted these points below. Additionally, I have a few concerns about potential confounding effects that were not accounted for in their analyses.

Major comments:

1) Line 96: "We also included data from a further 5 studies (right side of Figure 1), that had previously been identified by us to include neutralisation data after boosting with variant modified vaccines in an earlier analysis and remained relevant for this analysis". Why didn't the current inclusion criteria re-capture these studies? What was different about them so that they were not captured with the criteria detailed above? This should be explained in the manuscript, especially if it is possible other studies were missed.

2) Line 117: "Where a bivalent vaccine contained multiple immunogens (e.g. Ancestral + BA.5 or early VoC + BA.1) we classified the vaccine immunogen to be that of the latest chronological

variant." It seems important to keep track of whether the vaccine was bivalent or not, as inclusion of an older variant with a new variant would be expected to modulate (potentially reduce) the response. Additionally, were all studies on BA.5 with a bivalent vaccine? It would help if the authors could perform an additional sensitivity analysis that includes as a variable whether the vaccine was with a monovalent of bivalent antigen and whether this influenced responses. If this cannot be done, this limitation should be discussed. Additionally, if there were comparisons where the available data was only for a bivalent vaccine, this should be mentioned.

3) From Table 1, the effect of an update was stronger for ancestral vs. early VoC and ancestral vs. BA.1 vs. compared to the later updates. However, based on primary infection antisera, the antigenic distance between early VoC and BA.1 and BA.1 and BA.5 are at least as large as between most ancestral vs. early VoCs. The authors have a paragraph on potential confounders (lines 192-202) and account for number of prior exposures. However, as the pandemic goes on, individuals had both more prior exposures and more exposures to antigenically diverse antigens. Could this help explain the odd finding in Fig. S3 of 4 prior exposures in comparison 4, where the old immunogen has higher titers after boost than the updated immunogen? The authors seemingly have not account for the increasing complexities of immune histories at the time each study was conducted. If possible, it would be helpful to try to evaluate not only how number and homogeneity of exposure (I assume this is also number of exposures) but likely exposure to antigenically diverse antigens affects boosting. If this cannot be done, it should be further discussed, with mention of any empirical studies that were able to account for known prior infection histories.

4) This concern about the effect of complex infection histories also applies to the section, Optimal frequency of updating booster immunogens. In this section, are the authors arguing that the data were clean enough to directly compare how each vaccine update compared to the others? Given how ubiquitous Omicron was in 2022, it seems likely that more people also had natural infection with Omicron by the time the bivalent booster was being evaluated. Is it possible to account for complex infection histories in this analysis? If not, the effect of additional natural exposures should be discussed in relation to this finding.

Points of clarification:

Line 220: "However, under 60% (16/27) of the studies we identified reported both pre- and post-boost titres necessary to calculate fold-rise in titre for the immunogen / variant combinations required)." I am slightly confused by this sentence, as all the analyses described above report difference in fold-rise. Does this mean in the analysis, "we repeated the above analysis on the absolute neutralisation titres to a future variant reported after boosting", you were able to use all 27 studies? It would help if this could be stated directly here.

Line 504: "Primary vaccination and three additional exposures) are actually lower than the titres against BA. 1 after 3 exposures (two-course primary vaccination and one boost) (191 vs 604) suggesting that protective titres against the circulating variant are remaining relatively constant over time." I am not following this sentence. How does this demonstrate that titers against the circulating variant are constant?

Minor comments:

Line 44: "The advantage of an updated to a vaccine immunogen therefore". Should read "update".

Line 123: dependant should be dependent.

There is something weird with the references. All the studies used for the model 1-28 are not introduced until much later, while introduction starts at ref. 29.

Line 561: "titres following boosting and is fit only to the data shown in that did not include a". shown in ___ that, should there be a reference to a figure here?

Reviewer #3 (Remarks to the Author):

The authors ask an important question: will updating a vaccine antigen improve increase levels of protective neutralising antibody over what would be achieved by boosting immune levels with a vaccine antigen than has not been updated. The authors refer to these as 'updated booster' versus 'old booster' but these could be referred to as updated vaccine antigen and ‘previous’ vaccine antigen. Suggest replacing the word ‘booster’ in the title with ‘vaccine’.

The analytic design as presented in Figure 2A would be clearer in tabular form, with columns for “previous immunogen(s)”, “updated immunogen” and “future variant [i.e. test antigen]”. Each row could identify the number of studies included in each comparison rather than as presented in Table 1.

The authors approach to evaluating relative immunogenicity of previous versus updated vaccine antigens against antigenically advanced or immune escape variants is novel. This approach represents an advance over immunogenicity studies that have compared neutralising antibody titres against vaccine components. Instead, the authors have identified and analysed post-vaccination antibody levels against variants that circulated after the participants were vaccinated. This is perhaps the most important question for vaccine-elicited antibodies—not how much vaccination increases antibody levels against vaccine-like or previously encountered viruses, but whether vaccine updates increase protection against future variant viruses.

Retrospective analysis of this question contributes to understanding vaccine protection and potentially predicting vaccine effectiveness. The authors’ statement in the Discussion (line 301) that these data could inform decisions about future vaccine updates is based on this understanding but needs clarification—relative immunogenicity data would only be available for antigenically advanced variants circulating at the time candidate vaccine immunogens are evaluated. Improved predictors of population susceptibility, protective immunity and vaccine response would be needed to inform decisions to update vaccine components.

The main findings are presented in two manners: 1) in the Abstract and Discussion, fold-rise ratios are equated with increased antibody levels against future variant viruses following receipt of an updated vaccine antigen compared to receipt of the past vaccine antigen; 2) in the Results (Line 273), authors present the boost in titre compared to the past vaccine antigen as percent increase in odds of protection. The former is clearer because protection is usually expressed as ratios below 1. Additional translation into actual increases in levels of protection would improve understanding of the concept.

What does 40% relative increase in titres mean for protection from disease (Line 265). Are the authors able to assess how is the 40% relative benefit in neutralisation titres distributes across baseline antibody levels—is the relative greatest for those with lowest baseline titres against future variants?

Fold-rise can be a misleading measure if increases plateau at high baseline titres, and tends to be highest among those with lowest pre-vaccine titres. This relationship could affect their findings. It is unclear how the authors deal with that in studies for which pre-vaccine titres were not available.

In lines 287-291, the authors provide a useful translation of relative benefit at 2 levels of baseline immunity--from 60% to 68% protection, or from 91 to 94% protection against severe disease. This is not well explained in the Methods or supplemental methods, but additional translations across a wider range of values may be appropriate—even as a figure panel. Could the authors use an approach similar to that used in the Khoury Nature Medicine 2021 paper to compare measured or hypothetical distributions of neutralising antibodies from the identified studies to illustrate how shifting the antibody concentrations higher would result in improved protection against immune-escape variant viruses?

Line 310—Breadth of protection, rather than broadly cross-reactive antibodies, may occur as eliciting new antibodies to variant specific epitopes, or boosting levels of pre-existing cross reactive antibodies. For example, including an ancestral stain and Omicron-variant strain in the bivalent

booster is an example of maximising both. How do the authors interpret relative benefit of updating vaccine antigen on breadth of protection?

REVIEWER COMMENTS

Reviewer #1 (Remarks to the Author):

Cromer et al. perform a meta-analysis of published studies to model the benefit of boosting with an updated COVID19-vaccine compared to a previous vaccine antigen on titers of neutralizing antibodies against future variants. They find that the boost in neutralizing antibody titers was on average 40% higher against a future variant when using a more advanced variant as booster compared to an older variant. This study is interesting and important to judge the need for COVID-19 vaccine updates.

• Abstract line 16/17 + Introduction lines 37-39: Decisions on vaccine updates are not only been made based on neutralizing antibody titers but also on estimates of vaccine efficacy..

The reviewer is correct that neutralising antibodies are not the only metric used in the decision to update a vaccine. We agree that current vaccine efficacy may help guide the need to update. However, since vaccine efficacy data for candidate novel immunogens is not available to help guide the choice of future immunogen, historically in vitro measures of neutralising antibody titres have played a significant role. We have inserted the word “largely” into the abstract and introduction at the positions indicated to be more inclusive as the reviewer suggests. The abstract text now reads:

> *“These updated antigens were chosen and approved based largely on in vitro neutralisation titres against recent SARS-CoV-2 variants.”*

And the Introduction text reads:

> *“As a result, the assessment and comparison of different updated vaccine immunogens has been made based largely on neutralising antibody titres after vaccination, most commonly against the vaccine immunogen”*

• Figure 2: Symbols are not explained.

We thank the reviewer for pointing this out. A legend has now been included for the symbols.

• N should be added to figure legends.

Figure 3 and Supplementary Figures 1, 2 and 3 have all been changed to include the number of subjects.

• Lines 164-172: This paragraph is a bit confusing. In Figure 2B not all of the 27 studies could be used as not all studies contained pre-boost titers. It might be helpful to already give here the info that for Figure B only 16 studies were used.

We agree with this suggestion by the reviewer and have modified the end of this paragraph to state:

"The fold-changes from the 16 studies that included pre-boost neutralisation titres are depicted in Figure 2B."

• Information in Table 2 might be difficult to understand for a broader audience. Some more explanation might be helpful.

We agree that additional explanation regarding the parameters in Table 2 may be helpful. We have modified table 2 as shown below:

Dependant Variable	Parameter	Value (95% CI), p-value	Interpretation
Fold rise in neutralisation titres after boosting	Intercept	1.245 (1.071-1.419), $p < 0.001$	Initial boost with an older immunogen gives a $10^{1.245}$ = 17.6 fold rise
	Immunogen (updated vs older)	0.145 (0.031-0.259), $p = 0.013$	Updated immunogen provides $10^{0.145}$ = 1.4 fold greater fold rise than older immunogen.
	Exposure number	-0.404 (-0.488--0.32), $p < 0.001$	Each additional prior exposure reduces the fold rise by $10^{0.404}$ = 2.5 fold.

Table 2
Parameter values and their interpretations for the mixed effects model described in Equation S2, that predicts the fold-rise in neutralisation titres following boosting and is fit to the data shown in Supplementary Figure 1A.

And have modified the text describing Table 2 as follows:

"Therefore, we added these factors as co-variates in our model, the results of which are presented in Table 2."

• Figure 3: Legend seems to be incomplete

We have now checked the legend and ensured that it is complete.

• Line 379: "COVID-19 variants" might be changed to "SARS-CoV-2 variants" as the authors talk here about virus variants and not disease. Alternatively, "COVID-19 vaccine variants" may be appropriate.

We agree with this suggestion and have chosen to replace the word "COVID-19" with "SARS-CoV-2" so that the text in this section now reads:

"Another major limitation of any retrospective study of SARS-CoV-2 variants and immunogenicity…"

• Some references seem to be incomplete such as ref. 29, 30, 31 etc.

The reviewer is correct that these references appear to be incomplete, and this is because they are reports or websites from policy bodies. We have updated the reference formatting for these references.

• Uncertainty in the number of prior infections may be a limitation of the modelling approach as the authors show that the increased benefit of an updated compared to a previous vaccine antigen becomes smaller after multiple exposures. The authors define infection history as 0 (uninfected) or 1 (infected) or the proportion of the cohort that had been infected. Multiple infections and especially the infecting variant and time since infection might have a big influence on fold-increase of neutralizing titers after booster. As many of these factors are unknown for the majority of studies it might be difficult to incorporate them. However, it may be good to add these limitations to the discussion.

The reviewer is correct that it would be difficult to include these factors into our model as such details are not reported by many of the studies. However, we agree that diversity in exposure may influence the rise in neutralisation titres obtained after boosting, and so have inserted a comment on this into the discussion.

The relevant section of the discussion now reads:

> "The impact of increased antigenic exposure, as well as of multiple exposures were not something it was feasible to interrogate with the available data, as most studies did not report on the variant by which subjects had previously been infected, nor on their number of prior infections. However one study[1] did report post boost neutralisation titres following either pre-omicron (cohorts iii and vi) or omicron (cohorts iv and viii) infection. In both cases neutralisation titles were higher when subjects had been boosted with an updated rather than an older immunogen."

Reviewer #2 (Remarks to the Author):

Cromer et al. have performed a systematic literature review and statistical analysis of data on the effect of vaccine updates on boosting neutralizing antibody titers to a future infecting variant of SARS-CoV-2. The study leverages the group's previous work establishing an association between neutralizing antibody titers and protection to then apply the modeling results here to interpretation of the effect of vaccine updates on improving protection against future circulating variants. The manuscript is overall well-written, and many of the sensitivity analyses are justified. There were some instances where I was not able to follow the authors line of logic and have noted these points below. Additionally, I have a few concerns about potential confounding effects that were not accounted for in their analyses.

Major comments:

1) Line 96: "We also included data from a further 5 studies (right side of Figure 1), that had previously been identified by us to include neutralisation data after boosting with

variant modified vaccines in an earlier analysis and remained relevant for this analysis". Why didn't the current inclusion criteria re-capture these studies? What was different about them so that they were not captured with the criteria detailed above? This should be explained in the manuscript, especially if it is possible other studies were missed.

Of the five studies that were not identified through our current inclusion criteria, one (Miller et. al. 2023) was only indexed as a preprint and had been published subsequently with additional data, one (Collier et al 2023) was omitted from View-Hub and three (Pajon, 2022, Launay, 2022 and Chalkias *Nat Med* 2022) included a beta adjuvanted vaccine as their "updated" vaccine - such experimental vaccines were not recorded in View-Hub.

We have now included a sentence in the manuscript to explain this. The sentence reads:

> *"We also included data from a further 5 studies (right side of **Error! Reference source not found.**), that had previously been identified by us to include neutralisation data after boosting with variant modified vaccines in an earlier analysis[38] and remained relevant for this analysis. Three of these additional studies included a beta containing vaccine[6,19,20], which was not indexed in View-Hub, and another two were either omitted[10], or only had the preprint version indexed in View-Hub[11] (and the published version was used in our analysis). Together this led to a total of 28 studies from which we could extract data relevant to this analysis (Figure 1)."*

2) Line 117: "Where a bivalent vaccine contained multiple immunogens (e.g. Ancestral + BA.5 or early VoC + BA.1) we classified the vaccine immunogen to be that of the latest chronological variant." It seems important to keep track of whether the vaccine was bivalent or not, as inclusion of an older variant with a new variant would be expected to modulate (potentially reduce) the response. Additionally, were all studies on BA.5 with a bivalent vaccine? It would help if the authors could perform an additional sensitivity analysis that includes as a variable whether the vaccine was with a monovalent of bivalent antigen and whether this influenced responses. If this cannot be done, this limitation should be discussed. Additionally, if there were comparisons where the available data was only for a bivalent vaccine, this should be mentioned.

We thank the reviewer for this important observation regarding different vaccine compositions. In response to their question regarding the BA.5 vaccine, we can confirm that the majority of BA.1 and BA.5 vaccines were bivalent vaccines that included the ancestral immunogen as well as the BA.1 / BA.5 immunogen. In some cases monovalent and bivalent vaccines were compared in the same study. For example, there were two publications in which data was reported from a BA.1 monovalent vaccine, one of which also reported data for a bivalent BA.1 / Delta vaccine and a bivalent BA.1 / Beta vaccine. In addition, one publication reported data on a combined XBB and BA.5 vaccine. So 'bivalent' did not always imply a combination of an updated with an ancestral vaccine.

In order to test the reviewer's question of whether using a bivalent vs monovalent vaccine immunogen influenced our results we have now performed a sensitivity analysis that included a "valency" variable to specify whether a vaccine contained a monovalent or bivalent immunogen. We found that this "valency" variable was not considered to be a significant variable in our model (p=0.44), and therefore have not included this in the manuscript.

3) From Table 1, the effect of an update was stronger for ancestral vs. early VoC and ancestral vs. BA.1 vs. compared to the later updates. However, based on primary infection antisera, the antigenic distance between early VoC and BA.1 and BA.1 and BA.5 are at least as large as between most ancestral vs. early VoCs. The authors have a paragraph on potential confounders (lines 192-202) and account for number of prior exposures. However, as the pandemic goes on, individuals had both more prior exposures and more exposures to antigenically diverse antigens. Could this help explain the odd finding in Fig. S3 of 4 prior exposures in comparison 4, where the old immunogen has higher titers after boost than the updated immunogen?

In this question the reviewer makes an important observation that In Supplementary Figure 3 it appears that the neutralisation titres against XBB after 4 prior exposures in some cases appear higher after the old vaccine than after the updated vaccine. However, it is important to realise that these measurements come from different studies which almost certainly use different assays. In the one study where we could make a direct comparison between the old and updated vaccine in using the same assay on more than 5 subjects there was actually an increase in neutralisation titre for the updated vaccine (darker solid line in comparison 4, 4 exposures panel of Supplementary Figure 3A). In addition, the fold rise in neutralisation titres (which we expect to be more comparable across studies) is higher for the updated vaccine than for the older vaccine for all comparisons after 4 exposures (Supplementary Figure 1A). Therefore, we do not feel that exposure to antigenically diverse antigens has directly impacted this comparison. However, in order to make it clearer that we have not directly accounted for antigenically diverse immunogens we have inserted the following into the discussion:

> "We attempted to account for these differences in our model by incorporating parameters to account for inter-study variation and prior exposure history, but we cannot rule out an additional unexpected impact of exposure to antigenically diverse immunogens that we did not fully capture."

The authors seemingly have not account for the increasing complexities of immune histories at the time each study was conducted. If possible, it would be helpful to try to evaluate not only how number and homogeneity of exposure (I assume this is also number of exposures) but likely exposure to antigenically diverse antigens affects boosting. If this cannot be done, it should be further discussed, with mention of any empirical studies that were able to account for known prior infection histories.

The reviewer makes an important point (also raised by reviewer 1) that we have not directly addressed how prior exposure to antigenically diverse variants may impact the

relative benefit of an old or updated booster. To address these comments by both reviewers 1 and 2, we have added a comment on this to the discussion section.

The relevant section of the discussion now reads:

> *"We might speculate how the ongoing circulation of and infection by antigenically distinct variants could affect boosting with different immunogens. On the one hand, recent infection or a high number of previous infections could decrease the rise in titres conferred by booster vaccination. This might occur if there is a 'maximum level of boosting', and the subject is near that maximum before vaccination. Alternatively, recent exposure to an immunogen similar to the one contained in the booster may also affect the level of boosting from the updated immunogen. The impact of recent antigenic exposure, as well as of multiple exposures were not something it was feasible to evaluate with the available data, as most studies did not report on the timing or variant with which subjects had previously been infected, nor on their number of prior infections. However, one study[1] did report post boost neutralisation titres following either pre-omicron (cohorts iii and vi) or omicron (cohorts iv and viii) infection. In both cases neutralisation titres were higher when subjects had been boosted with an updated rather than an older immunogen. Previous studies of the effects of prior infection and boosting in influenza have identified complex interactions[51], and similar studies should be considered to understand the effects of infection on boosting in COVID-19."*

4) This concern about the effect of complex infection histories also applies to the section, Optimal frequency of updating booster immunogens. In this section, are the authors arguing that the data were clean enough to directly compare how each vaccine update compared to the others? Given how ubiquitous Omicron was in 2022, it seems likely that more people also had natural infection with Omicron by the time the bivalent booster was being evaluated. Is it possible to account for complex infection histories in this analysis? If not, the effect of additional natural exposures should be discussed in relation to this finding.

We thank the reviewer for this comment. Indeed, in this section we are not arguing that the data conclusively estimate how each vaccine update compared to others. Rather we are commenting that for a later circulating variant, XBB (which is the only variant against which we can track the impact of multiple consecutive immunogen updates), the later boosters provide a greater fold rise in titres than the earlier boosters.

This is perhaps interesting in light of the reviewer's observation that more people had acquired natural omicron infection prior to receiving either the BA.1 or BA.5 bivalent booster. As we now comment in the discussion, one might expect that natural exposure to omicron infection would *reduce* the benefit of an omicron containing booster (as the subject has prior experience of the immunogen), or that it may *increase* the benefit due to priming of the immune system. In reality, we cannot determine the exact effect, as there is insufficient data available with this information provided.

We above expanded discussion on the potential effects of prior infection and diversity in exposure (detailed above) aims to now address these issues and limitations.

Points of clarification:

Line 220: “However, under 60% (16/27) of the studies we identified reported both pre- and post-boost titres necessary to calculate fold-rise in titre for the immunogen / variant combinations required).” I am slightly confused by this sentence, as all the analyses described above report difference in fold-rise. Does this mean in the analysis, “we repeated the above analysis on the absolute neutralisation titres to a future variant reported after boosting”, you were able to use all 27 studies? It would help if this could be stated directly here.

We apologise that this previous wording could have been confusing. We have modified lines 221 – 226 so that they now read:

> *“However, under 60% (16/27) of the studies that we identified reported both pre- and post-boost titres necessary to calculate fold-rise in titre for the immunogen / variant combinations required, and so only 16 studies contributed to the results presented above. Therefore, to confirm that the actual neutralisation titres were improved by updating the vaccine immunogen (and not just the fold increase in neutralisation titres), we repeated the above analysis on the absolute neutralisation titres to a future variant reported after boosting, which allowed us to incorporate the data from all 27 studies.”*

We have also modified the earlier section of the results, titled “Updated Immunogens Provide a Greater Boost to a Future Variant” to make it clear that data from only 16 studies are used in the early fold-rise analysis. The updated text now reads:

> “*We constructed a mixed effects linear regression model that accounted for between study heterogeneity and the differences in the sizes of the cohorts and incorporated data from 16 (of 27) studies that provided both pre and post boost neutralisation data. We found that updated antigens consistently predicted greater increases in neutralisation against the relevant variant.”*

Line 504: “Primary vaccination and three additional exposures) are actually lower than the titres against BA. 1 after 3 exposures (two-course primary vaccination and one boost) (191 vs 604) suggesting that protective titres against the circulating variant are remaining relatively constant over time.” I am not following this sentence. How does this demonstrate that titers against the circulating variant are constant?

We agree that the wording of this paragraph was unclear. We were attempting to highlight that absolute titres against the circulating variant may not be increasing over time, however we agree that the data are not clear enough to explain this given the very different assays used. For this reason we have now removed this paragraph from the manuscript.

Minor comments:

Line 44: “The advantage of an updated to a vaccine immunogen therefore”. Should read “update”.

This has been changed

Line 123: dependant should be dependent.

This has been changed

There is something weird with the references. All the studies used for the model 1-28 are not introduced until much later, while introduction starts at ref. 29.

This is because the studies appear in Supplementary Table 1, and is done to ensure the referencing in the table matches the referencing in the manuscript. We are happy to to adjust reference numbering according to journal style.

Line 561: “titres following boosting and is fit only to the data shown in that did not include a”. shown in ___ that, should there be a reference to a figure here?

We apologise that the reference to Supplementary Figure 1A was not included here. We have verified that it is included in the updated version.

Reviewer #3 (Remarks to the Author):

The authors ask an important question: will updating a vaccine antigen improve increase levels of protective neutralising antibody over what would be achieved by boosting immune levels with a vaccine antigen than has not been updated. The authors refer to these as 'updated booster' versus 'old booster' but these could be referred to as updated vaccine antigen and ‘previous’ vaccine antigen. Suggest replacing the word ‘booster’ in the title with ‘vaccine’.

We agree that the term “booster” and “vaccine” could both be used in the title of this work. The reason we have used the word “booster” in the title rather than “vaccine” is that we did not want to imply that this analysis related to primary course vaccination. We are concerned that replacing the word “booster” with the word “vaccine” may create a misunderstanding and have therefore elected not to edit the title. If the editor disagrees and would like the title changed we are happy to do this.

The analytic design as presented in Figure 2A would be clearer in tabular form, with columns for “previous immunogen(s)”, “updated immunogen” and “future variant [i.e. test antigen]”. Each row could identify the number of studies included in each comparison rather than as presented in Table 1.

We thank the reviewer for this suggestion. Indeed, we did consider multiple ways to represent the design that is now shown in Figure 2A, including both tabular and graphical form. It is our opinion that the graphical format is clearer and more intuitive, and can more easily be related to the data in Figure 2B. However, in response to the reviewer's suggestion, we have now also included this as a supplementary table (Supplementary Table 2) and referenced it in the text. The amended text now reads:

> "*We identified four such comparisons that could be made from the available data, and these are shown by the coloured lines in Figure 2A and are detailed in Supplementary Table 2.*"

And the supplementary table and caption are:

**Comparison No**	**Old Immunogen**	**Updated Immunogen**	**Future Variant**	**Number of contributing studies**
**Comparison 1 (Red)**	Ancestral	Early VoC	BA.1	15
**Comparison 2 (Blue)**	Ancestral	BA.1	BA.5	24
**Comparison 3 (Green)**	Early VoC	BA.1	BA.5	11
**Comparison 4 (Orange)**	BA.1	BA.5	XBB	15

Supplementary Table 2 Description of the comparisons made between different vaccine immunogens to induce neutralisation against future variants, and the number of studies with data that could contribute to each comparison.

The authors approach to evaluating relative immunogenicity of previous versus updated vaccine antigens against antigenically advanced or immune escape variants is novel. This approach represents an advance over immunogenicity studies that have compared neutralising antibody titres against vaccine components. Instead, the authors have identified and analysed post-vaccination antibody levels against variants that circulated after the participants were vaccinated. This is perhaps the most important question for vaccine-elicited antibodies—not how much vaccination increases antibody levels against vaccine-like or previously encountered viruses, but whether vaccine updates increase protection against future variant viruses.

We thank the reviewer for their supportive comment.

Retrospective analysis of this question contributes to understanding vaccine protection and potentially predicting vaccine effectiveness. The authors' statement in the Discussion (line 301) that these data could inform decisions about future vaccine updates is based on this understanding but needs clarification—relative immunogenicity data would only be available for antigenically advanced variants circulating at the time candidate vaccine immunogens are evaluated. Improved predictors of population susceptibility, protective immunity and vaccine response would be needed to inform decisions to update vaccine components.

Indeed the reviewer is correct in recognising at the time of decision making on the vaccine updates immunogenicity data would only be available on circulating variants *up to that chronological time point*, and that relative immunogenicity data on variants that emerge after vaccine deployment would not be available. In this work we have attempted to address this by looking at past examples of how boosting with an updated immunogen impacted neutralisation titres against the future variant that *was seen* at the time of deployment. However, even with the insights gained from our work, we still acknowledge the need for increased information regarding population immunity and vaccine effectiveness in order to inform decisions. To address this concern we have now inserted the following text into the first and third paragraphs of the discussion:

First paragraph:

> "The rapid evolution of SARS-CoV-2 variants over time means that decisions on updating of booster immunogens must always be made with imperfect information. In particular, decisions on which variant antigen to incorporate into a booster vaccine must be made when the future circulating variant (at the time the vaccine will be deployed) is unknown. Here, we have assembled the (limited) data currently available from past examples to inform this decision."

Third paragraph:

> "however, better prospective data collection on the current level of population immunity as well as the effectiveness of different booster regiments would add valuable information."

The main findings are presented in two manners: 1) in the Abstract and Discussion, fold-rise ratios are equated with increased antibody levels against future variant viruses following receipt of an updated vaccine antigen compared to receipt of the past vaccine antigen; 2) in the Results (Line 273), authors present the boost in titre compared to the past vaccine antigen as percent increase in odds of protection. The former is clearer because protection is usually expressed as ratios below 1. Additional translation into actual increases in levels of protection would improve understanding of the concept.

We address this comment together with the next 2 comments and below.

What does 40% relative increase in titres mean for protection from disease (Line 265). Are the authors able to assess how is the 40% relative benefit in neutralisation titres distributes across baseline antibody levels—is the relative greatest for those with lowest baseline titres against future variants?

In lines 287-291, the authors provide a useful translation of relative benefit at 2 levels of baseline immunity--from 60% to 68% protection, or from 91 to 94% protection against severe disease. This is not well explained in the Methods or supplemental methods, but additional translations across a wider range of values may be appropriate—even as a figure panel. Could the authors use an approach similar to that used in the Khoury Nature Medicine 2021 paper to compare measured or hypothetical distributions of

neutralising antibodies from the identified studies to illustrate how shifting the antibody concentrations higher would result in improved protection against immune-escape variant viruses?

As the reviewer has pointed out, in the manuscript we present the analysis of antibody titres as the increase in fold rises obtained from an updated as compared to an older booster. This format is used in both the Abstract, Discussion and first part of the results. In the second part of the results (the section to which the reviewer is referring) we attempt to relate how this fold rise in titres translates to an increase in protection. We report this as an increase in the odds of protection since the relationship between titres and protection is non-linear.

The reason for reporting the odds of protection is due to the underlying mathematical analysis from which the relationship is derived. It is actually somewhat complex to translate this increased odds of protection into a fixed increase in the level of protection. This is because, for any given increase in the odds of protection, the actual increase in protection depends on the background level of immunity in the population (this is a mathematical effect of how odds versus actual protection relate to each other). This is why we later state - for a specified level of prior immunity - what the increased protection would be (lines 333-339 in the updated manuscript). We then generalise this to state that for a range of protection after old booster vaccination, what the range of protection for updated booster vaccination would be (lines 341-346 in the updated manuscript).

The reviewer asks whether we could represent how this played out for different levels of immunity (rather than just give a range). We have taken the reviewers suggestion here (and below) and have included an additional figure (Supplementary Figure 4) that shows the improvement in protection for a range of values of old-booster immunity.

We hope that this answers the reviewer's question around the relative benefit across baseline antibody levels. In a head-to-head comparison of old vs. updated boosters, there would be higher observed protection at higher levels of old booster protection.

This is referenced in the text in two places, as indicated below.

> "We must therefore consider the predicted advantage of deploying an updated booster over and above the protection that would be achieved using an older booster vaccine. This is shown for a range of values in Supplementary Figure 4. If an older booster vaccine gave rise to 60% protection in the population against symptomatic disease and 91% protection against severe disease arising from the circulating variant (compared to a naïve population, dashed line in Supplementary Figure 4)…"

And

“More generally, if we assume that an older booster offers between 30% and 80% protection against symptomatic disease arising from the circulating variant (compared to a naïve population, grey shaded area in Supplementary Figure 4),…”

Supplementary figure 4 appears as:

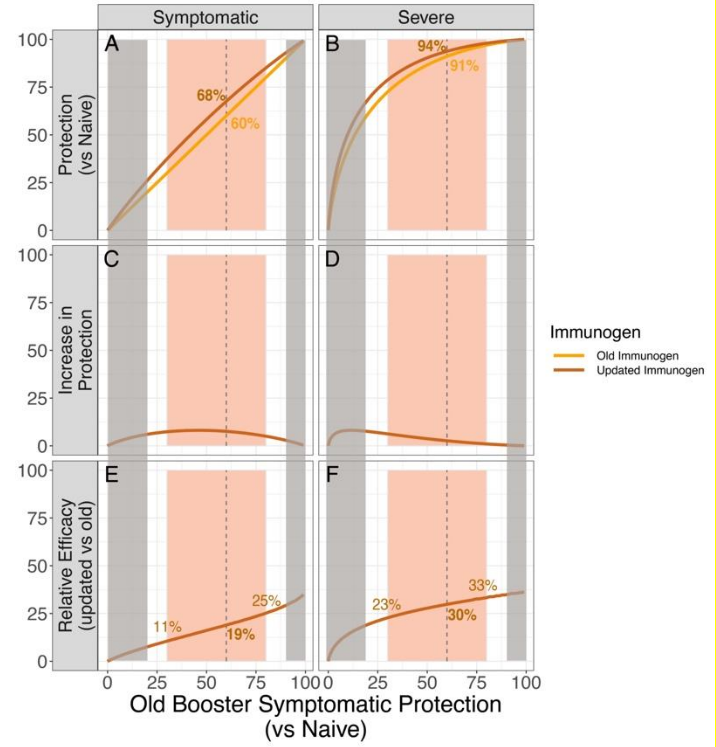

Supplementary Figure 4 *Impact of a vaccine containing the updated immunogen that confers a 40% increase in neutralising antibody titres on protection from symptomatic (A,C,E) and severe disease (B,D,F). The x-axis shows varying levels of protection against symptomatic disease arising from the circulating variant that would be conferred by using the older immunogen (as compared to a naïve individual). Panels A and B show absolute efficacy of the old and updated boosters (compared to a naïve population). Panels C and D show the absolute improvement in efficacy (for an updated immunogen compared to an older immunogen) and Panels E and F show the relative efficacy that would be obtained in a clinical trial directly comparing the old and updated immunogens. Grey dashed line shows the 60% protection level example discussed in the manuscript. Red shading shows the 30-80% range of old booster protection discussed in the manuscript. Numbers on the figure show percentages referenced in the text. We note that “old booster protection” of under 20% (representing an ineffectual boost) and “old booster protection” of greater than 90% (representing a boost that is as effective as the original vaccines against a non-escaped variant) are not realistic. These areas are shaded in grey and should be interpreted with caution.*

Fold-rise can be a misleading measure if increases plateau at high baseline titres, and tends to be highest among those with lowest pre-vaccine titres. This relationship could affect their findings. It is unclear how the authors deal with that in studies for which pre-vaccine titres were not available.

In this comment we think the reviewer refers to the frequent observation in influenza – that individuals with higher initial antibody levels show a lower fold-rise in antibody

levels after vaccination compared to people with low initial antibody levels. We assume this may be part of the reason that we see a decrease in the overall level of boosting with increased number of prior exposures (Figure S2). However, our analysis demonstrates that although the overall level of boost may decline with increased exposure, the improvement from using an updated immunogen seems more constant (Figure S1).

We have added additional text to the supplementary methods to make this clear. The relevant section now reads:

> "We observed a significant decrease in the magnitude of booster effect (for all variants) with increasing number of prior exposures, however, the estimated benefit of the updated immunogen remained constant across the number of prior exposures (Supplementary Figure 2**Error! Reference source not found.**)."

Line 310—Breadth of protection, rather than broadly cross-reactive antibodies, may occur as eliciting new antibodies to variant specific epitopes, or boosting levels of pre-existing cross reactive antibodies. For example, including an ancestral stain and Omicron-variant strain in the bivalent booster is an example of maximising both. How do the authors interpret relative benefit of updating vaccine antigen on breadth of protection?

This is an interesting question posed by the reviewer, and one which certainly warrants further investigation. However, there is no standard definition of 'breadth', and many approaches to breadth rely on the relative neutralisation of past variants. In this work we have shown that updating the vaccine immunogen increases neutralisation of the future circulating variant. It would certainly be both interesting and important to uncover whether this neutralisation advantage continued to variants even further into the future. However, the data to support this are unavailable, and the question beyond the scope of this analysis. The mechanisms behind the improved recognition of variants and whether this is related to breadth or strength of recognition is a question that would need to be addressed through further experimental studies. We have now included a comment on this in the discussion. The relevant section now reads:

> "Therefore, future studies should assess whether the relative benefit of updating a booster immunogen are related to either the breadth or the magnitude of the protection conferred and whether they can be predicted based on the antigenic relationships between the vaccine immunogen and the subsequent circulating variants."

REVIEWERS' COMMENTS

Reviewer #1 (Remarks to the Author):

Thank you for addressing all my comments in an appropriate manner. This is a very interesting and important study.

Reviewer #2 (Remarks to the Author):

Overall, the authors have addressed my questions and concerns. I do think it would be useful to report the valency sensitivity analysis in the manuscript, even though it was not significant, as I expect other readers will also have this question. Otherwise, I have no further comments.

Reviewer #3 (Remarks to the Author):

Thoughtful revision and response. Supplemental Figure 4 is a nice visual to explain relationship between protection and relative efficacy.
